# Differences in vaping topography in relation to adherence to exclusive electronic cigarette use in veterans

**DOI:** 10.1371/journal.pone.0195896

**Published:** 2018-04-25

**Authors:** Andrea Guerrero-Cignarella, Landy V. Luna Diaz, Kira Balestrini, Gregory Holt, Mehdi Mirsaeidi, Rafael Calderon-Candelario, Philip Whitney, Matthias Salathe, Michael A. Campos

**Affiliations:** 1 Division of Pulmonary, Allergy, Critical Care and Sleep Medicine, University of Miami Miller School of Medicine, Miami, Florida, United States of America; 2 Miami Veterans Affairs Medical Center, Miami, Florida, United States of America; Legacy, Schroeder Institute for Tobacco Research and Policy Studies, UNITED STATES

## Abstract

**Background:**

Understanding vaping patterns of electronic cigarette (EC) use is important to understand the real-life exposure to EC vapor. Long term information on vaping topography in relation to tobacco cigarette (TC) smoking cessation success has not been explored.

**Methods:**

Observational non-blinded study where active TC smokers were asked to replace TC with EC over 4 weeks (replacement phase, RP) followed by exclusive EC use for an additional 12 weeks (maintenance phase, MP). TC use and EC compliance was monitored weekly. Subjects were classified as success or failure whether or not they completed the protocol. Vaping information was stored and downloaded directly from the EC device and averaged per calendar day for analysis.

**Results:**

From 25 subjects that followed the protocol, sixteen succeeded in completing the RP and 8 the MP (32%). No significant differences in baseline characteristics were noted between subjects in the success and failure groups including markers of nicotine addiction, plasma cotinine levels or smoking history. Success subjects showed significantly longer puff duration (seconds per vape) and total overall vapor exposure (number of vapes x average vape duration or vape-seconds) in both study phases. Furthermore, subjects in the success group continued to increase the number of vapes, device voltage and wattage significantly as they transitioned into the MP. After an initial drop, subjects in the success group were able to regain plasma cotinine levels comparable to their TC use while subjects in the failure group could not. Cotinine levels significantly correlated with the average number of daily vapes and vapes-seconds, but not with other vaping parameters.

**Conclusion:**

The topography of smokers who adhere to exclusive EC use reflects a progressive and dynamic device adaptation over weeks to maintain baseline cotinine levels. The higher inhaled volume over time should be considered when addressing the potential toxic effects of EC and the variable EC adherence when addressing public health policies regarding their use.

## Introduction

With the widespread campaigns and policies to spread knowledge about the harms of tobacco cigarette (TC) smoking, the use of electronic nicotine delivery devices has gained popularity and become an emerging public health issue. In particular, the use of electronic cigarettes (EC) expanded significantly by attracting young groups. Between 2011–2016, the use of electronic cigarettes (EC) increased from 1.5% to 11–16% among high school students, and from 0.6% to 5.3% among middle school students [1]. In adults however, despite its perceived nature as a less dangerous alternative or potential to be used as a bridge to quit TC smoking, this proportion has not been expanding as rapidly [2]. Overall, 15.4% of adults in the US aged ≥18 years had ever used an EC while only 3.2% currently used EC in 2016 [3]. In the UK, where only 5.6% of adults reported to be current EC users during the same year, a plateau in EC use was observed since 2014 particularly among current TC smokers [4, 5]. In a UK survey, the most common reasons for TC smokers to use EC were to help them quit entirely or reduce the amount of tobacco smoked, while the main reason to stop EC use was because they didn’t feel like smoking a cigarette or they didn’t help deal with the cravings to smoke [5].

Nevertheless, the widespread use of ECs is worrisome in view of the many uncertainties related to their risk and abuse potential as they not only deliver nicotine but contain other constituents that are aerosolized as well, including low levels carcinogens and ultrafine particles that can increase risks for cardiovascular and other disease states [2]. Whether these devices are considered “less harmful” than TC or whether they play an important role as a smoking cessation tool is an ongoing debate. However, despite these reservations some major organizations now recommend smokers to replace TCs with ECs [6–8].

The variable epidemiologic trends of EC adherence as well as their potential risks highlights the importance to gain information about the vaping characteristics of their users, including assessment of the factors that influence their vaping patterns [9]. Topography refers to the smoking behavior of subjects in relation to how the device is used. Information regarding EC topography is starting to be reported in the literature. It is known that TC smokers modify their smoking behavior when switching to other cigarette types or different nicotine delivery devices [10, 11]. However most of these observations are based on either short term studies or took place in a clinical laboratory. Still, it was noted that smokers puff longer but slower when using ECs and adapt to the device with a learning curve that improves with experience. However, it is unclear whether these patterns occur in a less controlled environments or if they are sustained for longer periods of time. For this reason, a call for the need to measure EC topography in the natural environment has been recently issued [12].

There is a need to expand our knowledge of EC use behaviors in more real-life scenarios in order to better assess their toxicity and/or potential use as smoking cessation tools [13, 14]. Here we report the EC topography characteristics of smokers attempting to transition from TC smoking to EC vaping over several weeks.

## Methods

The study protocol was reviewed and approved by the Miami Veterans Affairs Medical Center Institutional Review Board. The trial was registered in clinicaltrials.gov (NCT 03251053). The protocol for this trial and supporting TREND checklist are available as supporting information; see S1 Protocol and IRB approval and S1 TREND checklist.

### Study participants

Active TC smokers were recruited within the hospital’s premises using local advertisement (posters and flyers) or from the pulmonary function laboratory. Inclusion criteria were age older than 18 years, willing to quit TC smoking, a smoking history of > 5 pack-years, and a normal baseline spirometry. We excluded subjects with any concomitant lung disease, prior thoracic surgery, HIV infection, chronic oral antibiotics or corticosteroids use within the last 3 months, active recreational drug use, regular or active vaping or inability to use EC.

### Study design

All participants were asked to complete questionnaires detailing their smoking habits including standardized questionnaires to assess nicotine addiction (Cigarette Dependence Scale and the Fargestrom Test for Nicotine Dependence) and anxiety levels (Beck Anxiety Inventory, Beck Depression Inventory and the Hospital Anxiety and Depression Scale). Active smokers were asked to replace TC smoking with EC vaping over a 4-week period (replacement phase, RP) and then maintain exclusive EC use for 12 more weeks (maintenance phase, MP). Subjects who were still TC smoking by week #5 (beginning of MP) were considered “early failures” and were excluded from further participation in the study. Subjects who relapsed to TC smoking during the MP were considered “late failures” and were also excluded from further participation. Subjects who completed the entire 16-week protocol were considered “success”. TC smoking was monitored by weekly in-person assessments of exhaled CO (ExCO) using the Smokelyzer^**®**^ (Bedfont Scientific, Ltd.Kent, UK), and venous carboxyhemoglobin (%COHb) using the Cobas B221 system^**®**^ (Roche, Branchburg, USA). ExCO levels >6 ppm [15] and %COHb >1.6% [16] were considered markers of active TC smoking. Subjects who missed a study visit were considered to have withdrawn from the study and excluded from further participation. Study participants received monetary compensation for expenses and transportation only at completion of the RP and the MP.

### EC device and topography measures

The EC device used was the eVic Supreme^**®**^ (Joyetech, ShenZhen, China) with the capacity to store the user’s daily vaping topography parameters (date, time, voltage, wattage and duration of each inhalation). This information was downloaded weekly using the myVapors^®^ software available from manufacturer. The e-liquid dispensed consisted of 50%/50% w/v propylene glycol (PG) and vegetable glycerin (VG) with a nicotine concentration of a 12 mg/ml. We chose this concentration to mimic the nicotine content of one vapor puff with one TC smoking puff (a pack per day consists of 180 puffs or 14 mg of nicotine of smoking and would be equivalent to a 1.2 ml eVic^®^ cartridge with 12 mg/ml of nicotine that provides 180–200 puffs). No other forms of nicotine replacement were provided other than the administered in the e-liquid, neither specific drugs or psychological/behavioral approaches for smoking cessation.

### Plasma cotinine levels

Blood drawn every 2 weeks at study visits was centrifuged and plasma stored in 1 ml aliquots at -20°C. Plasma samples were subsequently assayed using the Abnova^®^ Cotinine ELISA Kit KA0330 following the manufacturer’s instructions.

### Data analysis

For this observational non-blinded study, the study population was grouped in terms of their adherence (or not) to exclusive EC vaping at the end of the RP (successes or early failures) and MP (successes or late failures). Inhalations recorded from the device, including number, duration, voltage and wattage per vape were grouped and averaged per calendar days. In addition, equivalent to the pack-year concept of TC smoking, the total amount of time vaped per day we assessed through a new parameter called “vapes-seconds” (daily number of vapes times the duration of each vape). Statistical comparisons were performed using repeated measures ANOVA with Bonferroni corrections for multiple comparisons and Student’s t-tests for comparison of values with normal distribution or Wilcoxon and Kruskal-Wallis Tests for non-parametric variables as appropriate. Associations between independent variables (demographic, baseline nicotine and topography parameters) and the dependent variable “success” (switching TC to EC) was assessed by calculating the crude odds ratio (OR) via a logistic regression model. A multivariable mixed effect model was then fitted in order to evaluate the independent effect of the selected variables. Candidate predictors with a value of p < 0.10 in the univariate analysis were accepted for inclusion in the multilevel multivariate analysis. Variables were removed from the model when the p-value exceeded 0.10 and were kept in the final model when less than 0.05. All analyses were performed using JMP^**®**^ (version 13.1.0, SAS Institute Inc., Cary, NC) and SPSS^®^ 21.0 statistical software (SPSS Version 21.0, Armonk, NY). Differences were considered significant if p-value was <0.05.

## Results

### Participant characteristics

**Fig 1:**
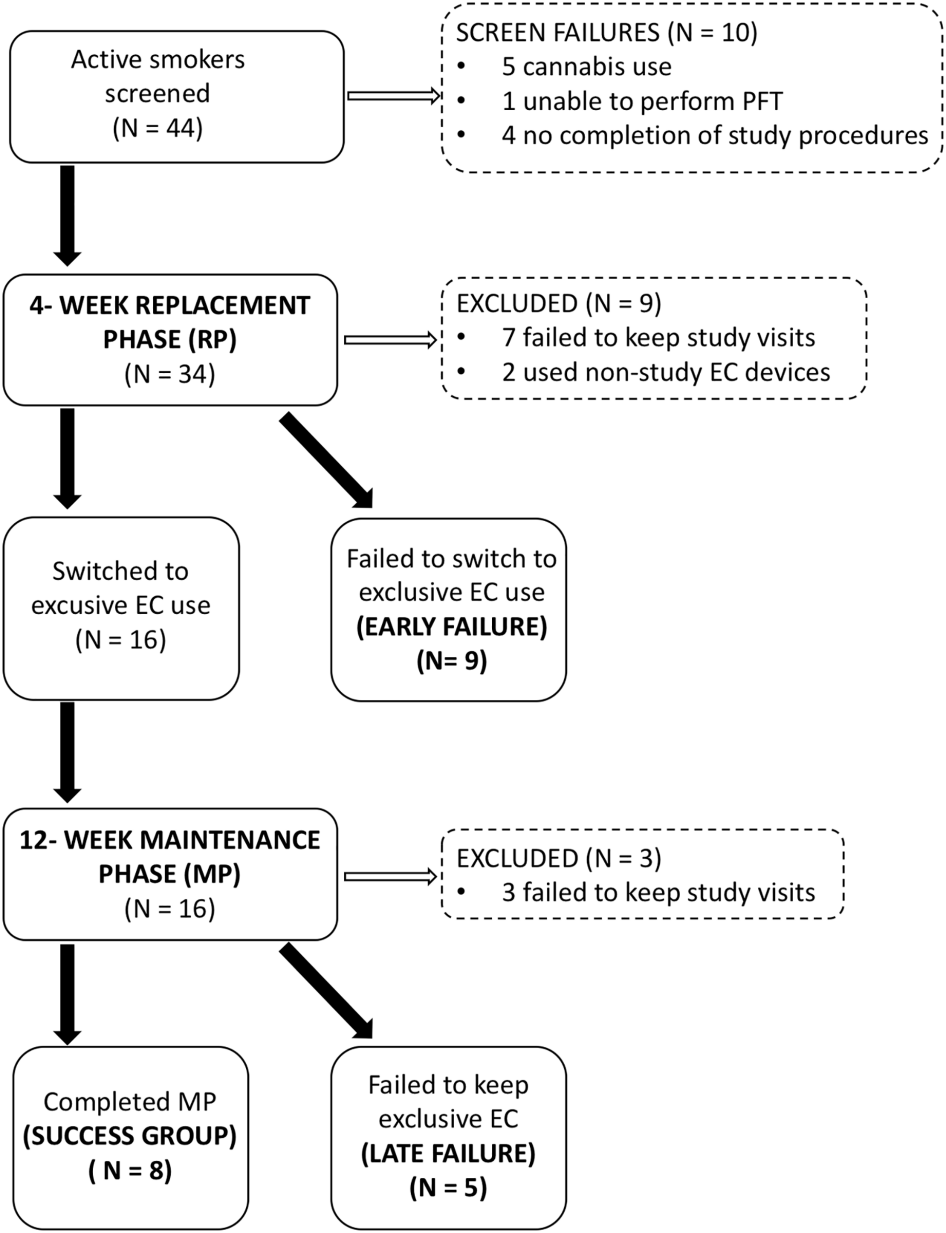
Flow diagram of the study design and study population. Active TC smokers entered a 4-week replacement phase in order to switch completely from TC to EC use. Subjects who succeeded entered the maintenance phase of exclusive EC for 12 weeks. PFT: Pulmonary function tests; TC: tobacco cigarette; EC: electronic cigarette.

The study flow chart and the number of subjects who entered each study phase are shown in Fig 1. From the 44 active smokers initially screened, 10 met exclusion criteria and 9 were excluded early in the replacement phase due of study violations or failure to keep up with weekly visits. Twenty-five subjects were successfully enrolled in the study protocol. From these, 16 subjects (64%) succeeded in replacing TC with EC over the initial 4-week period (replacement phase) and 8 subjects (32%) were able to maintain exclusive EC use for 12 additional weeks (maintenance phase).

Subjects who failed to switch to EC (early failures) or failed to maintain exclusive EC use during the MP (late failures) were grouped altogether as the failure group in order to compare their demographic and clinical characteristics with the success group. These comparisons are shown in Table 1. No significant differences were observed between the success and failure groups regarding age, gender, race, TC smoking history, baseline cotinine levels, and assessments of anxiety, depression, PTSD or scales of nicotine dependence. However, it was observed that subjects that failed during the RP had significantly higher anxiety scores compared with subjects who successfully made the switch to EC (S1 Table). Per inclusion criteria all participants required normal spirometry. However, subjects in the success group had significantly higher FEV_1_ values.

**Table 1:** Baseline demographic characteristics.

N		ALL	FAILURE GROUP^†^	SUCCESS GROUP	*p*
		25	17	8	
Age, years		57.8 ± 5.3	59 ± 6	56.3 ± 4.1	0.29
Gender, %male		96	90	100	0.33
Race %	African American	80	64.3	87.5	0.21
	Hispanic	10	21.4	0	
	Caucasian	10	14.3	12.5	
**Smoking history**					
Pack-years		49.8 ± 31.4	54.2 ± 35.8	43.2 ± 26.3	0.46
# Cigarettes per day		15.1 ± 8.6	17.5 ± 10.3	12.1 ± 6.6	0.22
**Plasma cotinine levels, ng/ml**		499.6 ± 256.8	533.5 ± 242.5	440.3 ± 286.5	0.42
**History of Mental Illness %**		94.7	90.9	100	0.28
**History of Drug Abuse %**		89.4	81.8	100	0.12
**Psychological Assessments**					
CDS		37.4 ± 16.5	30.54 ± 17.14	44.2 ± 13.9	0.07
FTND		5.8 ± 1.6	6.23 ± 1.78	5.5 ± 1.9	0.39
BAI *		5 (0–18.7)	7 (0.75–13.75)	7 (0.5–25)	0.96
BDI *		74.5 (0–24.5)	4.5 (0.25–22.5)	10.5 (0–30.7)	1.00
HADS A *		5 (0.5–9)	8 (3–9)	3.5 (0.25–12.5)	0.86
HADS D *		3 (1–7.5)	3 (1–9)	3.5 (0–14)	1.00
PTSD		92.1 ± 31.2	92.5 ± 19.49	96 ± 39.26	0.85
**Pulmonary Function**					
FEV_1_ (L)		2.9 ± 0.4	2.7 ± 0.43	3.18 ± 0.3	0.02
FEV_1_%		78.7 ± 10.5	77.6 ± 14.5	81.2 ± 6.2	0.52
FEV_1_/FVC		76 ± 7.1	76.8 ± 7.8	73.8 ± 6.1	0.37
FEF25-75%		69.6 ± 21.6	69.9 ± 27.7	68.3 ± 14.1	0.88

Comparison of characteristics between subjects that failed or succeed to switch TC with EC as outlined in the protocol. Results are expressed as Mean ± standard deviation.

*Nonparametric variables: Median (IQR) (Wilcoxon/Kruskal-Wallis Test)

^**†**^Includes subjects in the early failure (N = 9) and late failure (N = 8) groups.

CDS: Cigarette Dependence Scale, FTND: Fargestrom Test for Nicotine Dependence, BAI: Beck

Anxiety Inventory, BDI: Beck Depression Inventory, HADS A: Hospital Anxiety and Depression Scale

Anxiety, HADS D: Hospital Anxiety and Depression Scale Depression, PTSD: Post Traumatic Stress

Disorder, FEV_1_: Forced Expiratory Volume in One Second, FVC: Forced Vital Capacity ratio,

FEF: Forced Expiratory Flow.

**Fig 2:**
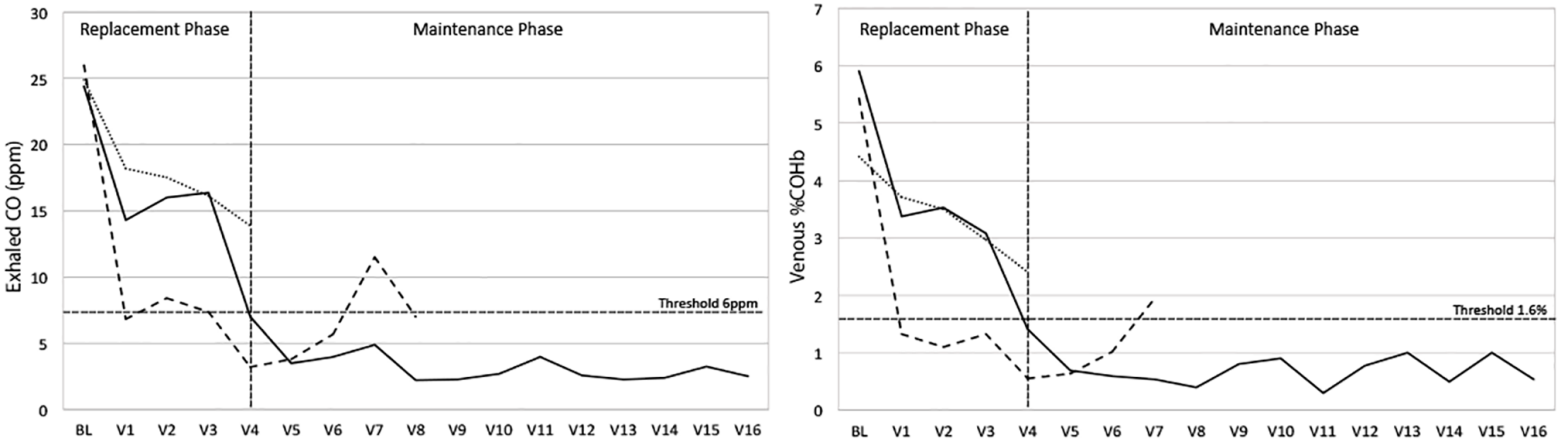
Monitoring of tobacco cigarette use. Mean exhaled CO and venous carboxyhemoglobin (%COHb) was monitored during each study visit. Levels of exhaled CO >6 ppm and %COHb >1.6% were considered as indicators of active TC use. Subjects were grouped as early failures (dotted lines, N = 9) if they continued to smoke TC by the end of the replacement phase and late failures (dashed lines, N = 5) if they relapsed during the maintenance phase. 8 subjects completed the maintenance phase (continuous line).

The trends in exhaled CO levels (ppm) and venous %COHb values used to monitor TC smoking and define the success and failure groups are shown in Fig 2. We observed that subjects in the success group had a more gradual decline in both ExCO and %COHb during the RP compared with subjects who subsequently became late failures.

### Vaping topography

**Fig 3:**
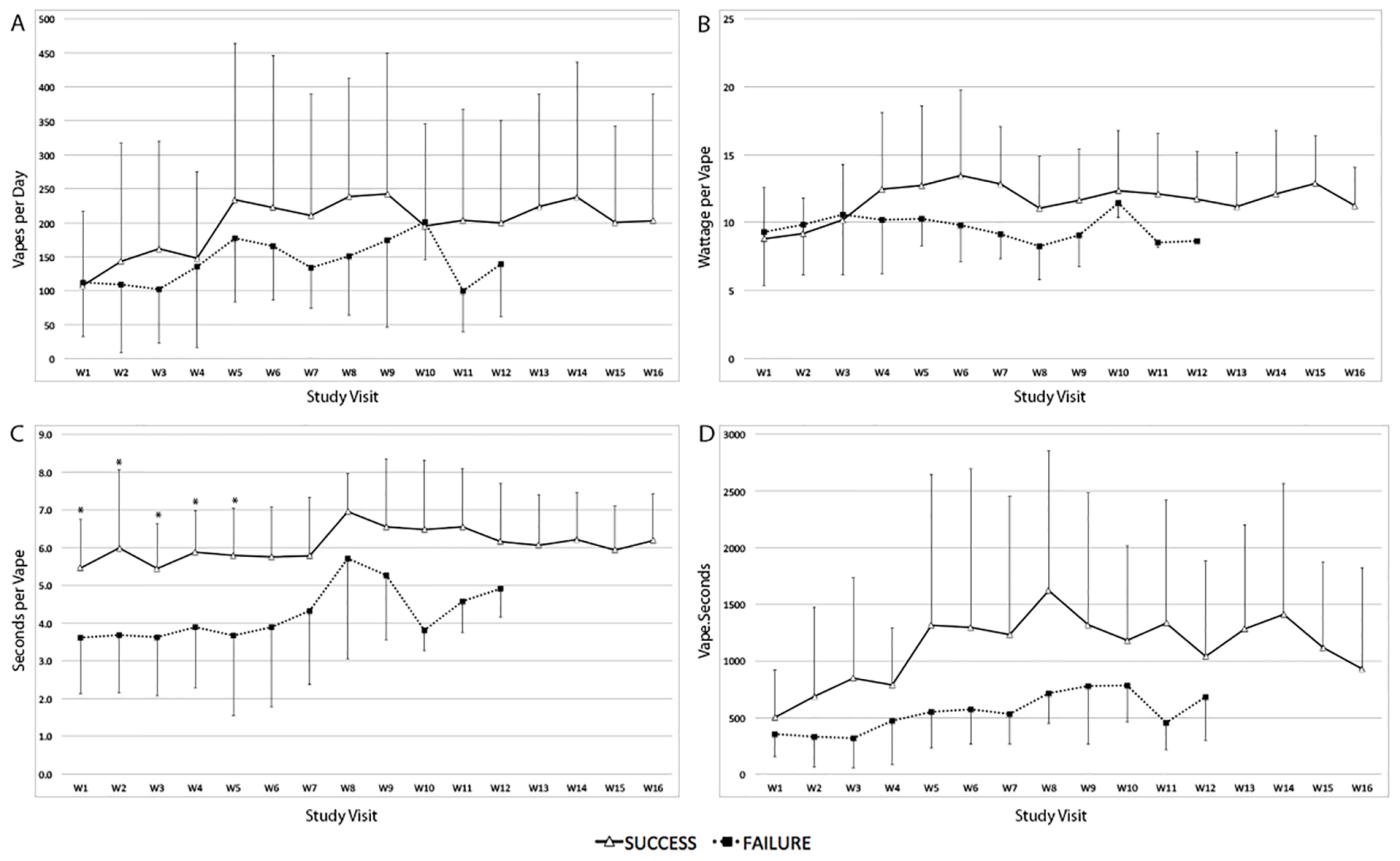
Vaping characteristics of subjects who failed (black squares) or succeeded (white triangles) replacement of tobacco smoking to electronic cigarette use. **A**. Average number of vapes per day. **B**. Average EC wattage used per vape. **C**. Average seconds per vape. **D**. Average vape-seconds (number of vapes times duration of each vape in seconds).

The weekly average values of the different vaping topography parameters assessed during the study for both the success and failure groups are shown in Fig 3 and the average values of these parameters per phase per group are summarized in Table 2. Although there were no statistical differences in the number of daily vapes between the success and failure groups, the success group had significantly longer durations per puff (seconds per vape) and overall inhalation time (vape-seconds). This was noted in both the RP and MP. Furthermore, subjects in the success group were able to further increase the number of vapes, device voltage and wattage in a significant way as they transitioned into the maintenance phase (S2 Table). Subjects in the failure group only significantly increased the number of vapes a day but in a lower magnitude compared to the success group.

**Table 2:** Vaping topography parameters of the success and failure groups by phases.

		Success Group	Failure Group	*p*
**Vapes per Day**	Replacement Phase	139.4 ± 138.0	114.6 ± 94.0	0.32
	Maintenance Phase	218.0 ± 173.3	159.9 ± 76.7	0.10
**Voltage per Vape**	Replacement Phase	3.85 ± 0.76	3.69 ± 0.77	0.38
	Maintenance Phase	4.32 ± 0.78	3.68 ± 0.43	<0.01
**Wattage per Vape**	Replacement Phase	10.0 ± 4.14	9.9 ± 3.9	0.78
	Maintenance Phase	12.1 ± 4.2	9.4 ± 2.0	<0.01
**Seconds per Vape**	Replacement Phase	5.7 ± 1.4	3.7 ± 1.5	<0.01
	Maintenance Phase	6.1 ± 1.3	4.4 ± 1.9	<0.01
**Vape-Seconds**	Replacement Phase	698.7 ± 651.9	367.5 ± 284.0	<0.01
	Maintenance Phase	1259.7 ± 1037.8	622.3 ± 295.8	<0.01

#### Replacement phase analysis

During this phase, the early failure group had statistically significant lower number of vapes, EC voltage, duration of vapes and vape-seconds compared to subjects who successfully switched to EC within the first 4 weeks (S1 Fig). Subjects who completed the replacement phase but subsequently became late failures, already exhibited a significant lower vape duration compared with subjects who succeeded both phases (3.34 s ± 1.96 vs 5.7 s ± 1.4, p<0.0001) (S2 Fig).

#### Maintenance phase analysis

As subjects entered the maintenance phase, those in the success group further increased the number of daily vapes (from 139 ± 138 to 218 ± 173.3, p = 0.02) while late failure subjects did not (160 ± 98 to 159.9 ± 76.7, NS). Similarly, late failure subjects did not show significant increases in EC wattage, voltage, duration per vape or vape-seconds as subjects in the success group did (data not shown).

Vaping patterns did not correlate significantly with TC smoking features such as pack-years, number of TC smoked per day at study entry nor the degree of nicotine dependence as measured by standardized questionnaires (data not shown).

### Vaping and cotinine levels

**Fig 4:**
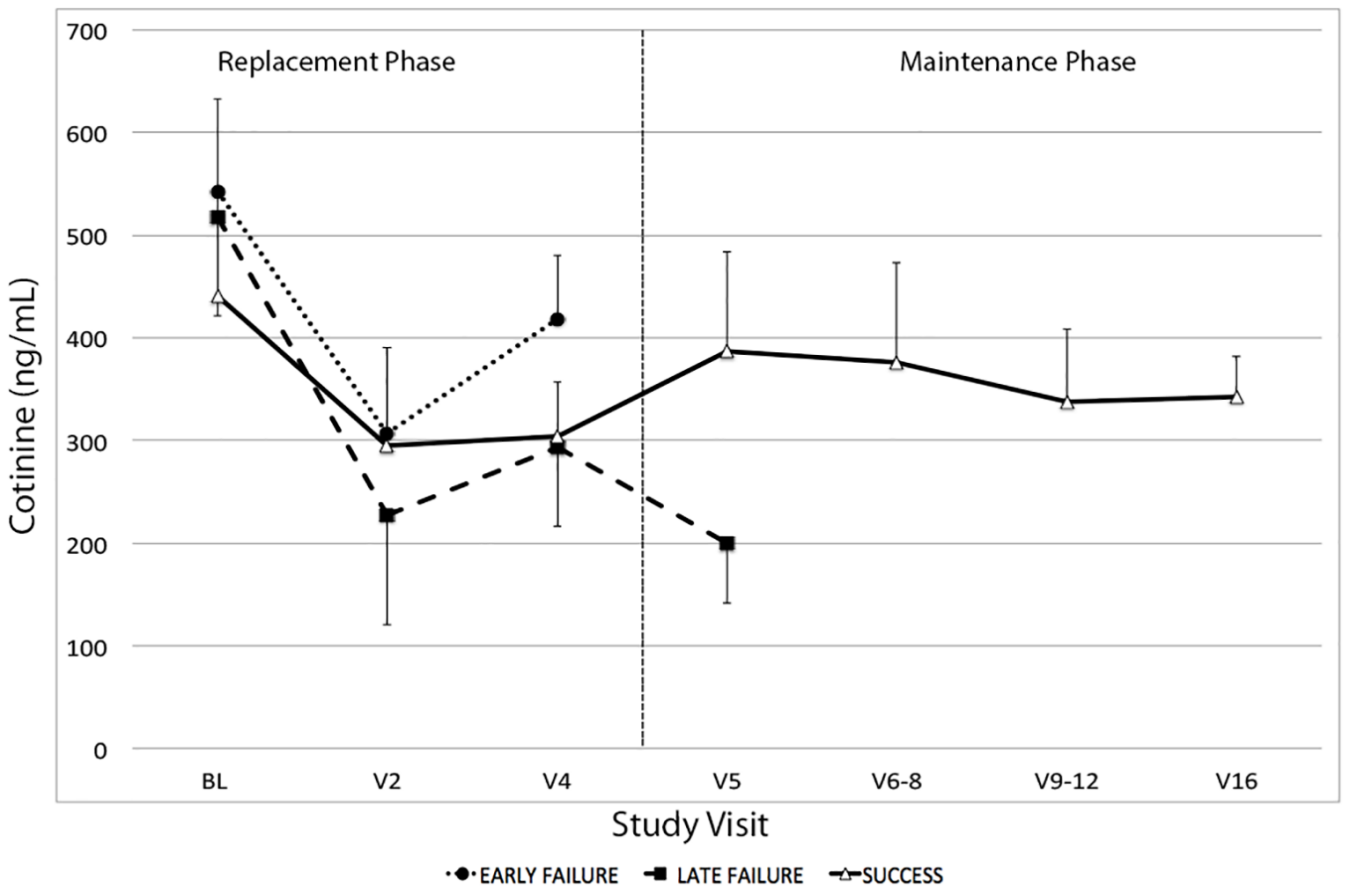
Average weekly plasma cotinine levels in subjects who succeeded or failed conversion of tobacco smoking to electronic cigarette vaping during the study. Plasma samples were collected at each study visit. Cotinine values were measured by ELISA. For late failure subjects, values are shown up to the week before they relapsed to TC use.

There were no differences in baseline cotinine levels between subjects in the success and failure groups upon study entry while smoking TC. During the replacement phase, cotinine levels dropped in all subjects as they tried to adapt to EC use (Fig 4). However, subjects in the success group were able to regain baseline cotinine levels. By week 5 (beginning of the maintenance phase) they already had similar plasma cotinine levels compared to baseline values (387.1 ± 255.7 ng/ml vs. 440.3 ± 286.1 ng/ml, respectively, p = 0.88). Likewise, their cotinine levels at the end of the trial (week 16) were similar to baseline values (337.3 ± 202.2 ng/ml vs. 440.3 ± 286.5 ng/ml, p = 0.058). On the contrary, subjects in the failure group had significantly lower plasma cotinine levels (in samples drawn in the visit prior to the relapse) compared to baseline values (352.0 ± 197.4 vs. 533.5 ± 242.8, respectively, p = 0.04).

Finally, we performed a regression analysis of variable associated with the outcome “success” (switch from TC to EC) for the replacement phase. We included race, CDS, baseline cotinine level and average EC topography variables (average number of daily puffs, voltage, wattage and puff-seconds) as independent variables of significance. As noted in Table 3, the only topography parameter associated with success of TC to EC switch was inhalation duration with an OR of 3.98 (1.41–17.50).

**Table 3:** Mixed effect regression analysis of variables associated with success in switching TC to EC over 4 weeks.

Variable	P-value	OR (95% CI)
Cigarette Dependence Scale score	**0.04**	**1.38 (1.01–2.03)**
Cotinine BL	0.16	1 (0.984–1.004)
Average puffs per day	0.43	1.02 (0.964–1.087)
Average voltage	0.29	2.38 (0.23–24.39)
Average wattage	0.36	1.53 (0.591–3.97)
Average inhalation duration (sec)	**0.014**	**3.98 (1.41–17.50)**
Average puff-seconds	0.33	1.01 (0.99–1.02)
Race (African American)	0.36	2.35 (0.05–134.73)

## Discussion

This study compares specific puffing topography changes that occur over several weeks in smokers in relation to switching exclusively to EC vaping. This success depends on a compensatory response that is mostly characterized by significant increases in inhalation time per puff and total inhalation time per day (vapes-seconds). As opposed to subjects who failed, subjects who could switch completely to EC vaping were able to achieve similar plasma cotinine levels compared to their previous habit smoking TC. To the best of our knowledge, this study is unique in that it provides information regarding different vaping topography patterns in relation to EC vaping adherence recorded in a real-life scenario and for an extended observation time period.

Even though we did not study our population’s baseline topography features while smoking TC, our results can be placed in context with other studies that evaluated the topography changes that occur when transitioning from TC smoking to EC vaping. While it has been described that puffing variables are not significantly affected by changing the nicotine content of TC [17], they may change when transitioning to a different device. In a shorter duration study, Lee et al. evaluated the puffing behavior of twenty smokers naïve to EC who switched to a first-generation EC for two weeks [10]. They found that after one week of using EC, participants significantly increased the average time per puff from 2.2 ± 0.1 to 3.1 ± 0.3 s and that this value remained the same by the second week. The authors noted that their limited observation time could not predict long-term adherence to the device. Farsalinos et al. also described that EC use in subjects undergoing short (5 and 20-minute) observations requires longer inhalation times compared to conventional cigarettes (4.7 s versus 2.1 s respectively) [18], an observation similar to what was also reported by others [11, 19, 20]. It should be noted that in most of these studies, nicotine delivery by the EC device was not standardized and behavioral compensation has been described when smokers switch to EC devices with different nicotine contents [21–23]. To minimize this potential confounder, we tried to mimic the “per puff” nicotine content of our EC device with that of a conventional cigarette. Our nicotine content estimate is based on the nicotine content of research grade cigarettes, namely 2R4F and 3R4F, made by the University of Kentucky (http://www.ca.uky.edu/refcig/). These cigarettes were promoted by the US Scientific Advisory Board of the Council for Tobacco Research to be standard for research use and constructed to represent typical segments of the American market (https://ctrp.uky.edu/resources/pdf/webdocs/Mainstream%20Smoke%20Chemistry%203R4F,%202R4F.pdf). We acknowledge from some reports that commercial TC have variable nicotine contents and many higher than what we calculated [24, 25]. Some have suggested that a 20 mg/ml nicotine is needed for delivery comparable to a conventional cigarette [18], but we estimated that the required nicotine concentration was lower. Nevertheless, an underestimation of nicotine in EC devices likely occurs in real-life and in this particular study, may be advantageous to highlight better the different topography patterns associated with adherence and adaptation. In this way, we observed that compliance to adhere to EC requires longer inhalation times starting immediately after switchinging to EC vaping and persisting as an ongoing process over several weeks.

The performance properties of different EC types are quite variable, with different aerosol density delivered from puff to puff, non-uniform nicotine delivery between different EC models and a variable amount of vacuum (suction) required among different EC brands [26–28]. Even over time, a variable amount of vacuum (suction) is required in order to maintain a constant aerosol production from the same EC [26]. This may be one of the reasons why our subjects in the success group continued to increase their puffing topography variables as they entered the maintenance phase, which reflects an ongoing adaptation that goes beyond the initial device transition period. We acknowledge that our observations are directly applicable only to the type of EC device used in this study, but overall our results further confirm the importance of device adaptation over time.

Unfortunately, we could not identify specific baseline subject characteristics that predict who can successfully replace TC smoking with EC vaping. ECs are more complex than cigarettes (due to the different components) and require familiarity to use. It has been described that the learning curve to maximize the nicotine delivery potential of ECs is more pronounced in experienced subjects, who end up using the device more intensively compared to naïve users [29, 30]. This explains why EC-naïve users exhibit lower serum nicotine levels compared with experienced users [30–33]. Although we did not include experienced EC users, it is possible that baseline topography differences may have influenced success rates as considerable topography variability between subjects smoking both conventional cigarettes and EC has been reported [21, 34, 35]. Here we show how different topography patterns relate to EC vaping adherence over time.

The specific topography data on EC adherence provided here is relevant for the current debate regarding EC policies. While some are worried that making EC devices more available (as “harm reducing” devices) may lead to greater EC adoption and nicotine dependence, others suggest it may not become a major public health threat due issues with adaptability to the device [36]. Studies like ours suggest that probably the latter is more likely, as only a third of subjects (32%) were able to completely replace cigarettes to EC over 16 weeks, a proportion similar (42%) to what was reported in a shorter (72 h) cigarette to EC conversion trial on 38 subjects [11] and better (14 and 26%) than what was reported in two larger randomized clinical trials at 12 weeks [37, 38]. The proportion of individuals that have tried EC are 5 times more than the proportion of current EC users [5]. Failure to adapt to exclusive EC use may be due to different reasons, including lower cigarette craving score reductions [11] and lower levels of liking compared with TC smoking [20]. It is also possible that differences in nicotine delivery patterns influence EC adherence, as ECs appear to deliver nicotine following a more intermittent dosing pattern compared with a more bolus dosing pattern delivered by TC smoking [39]. Here we demonstrate that individual adaptation to the EC device over time correlates with maintaining baseline plasma cotinine levels.

One strength of our study is that puffing variables were downloaded directly from the EC device, revealing real-life vaping conditions. Prior studies have been performed in laboratory settings using indirect ways of measuring topography such as analyzing video recordings and online videos or attaching flow meters, mouthpieces or modified cigarette topography analyzers to the device which may influence real vaping topography characteristics [10, 11, 17–19, 27, 34, 40]. In addition, most of these studies were performed under the supervision of investigators or over a short period of time (from a few puffs, to hours or a few days) [26, 28, 32, 41]. In a study intended to measure vaping topography in the subject’s natural environment over 24h, Robinson et al. requested users to utilize a hand-held monitoring device for each puffing event and noted patterns not detected in shorter laboratory assessments [34]. We believe that the information downloaded directly from the device used in our study provides even more accurate real-time information. In this way, more accurate topographic assessment may enhance our estimations of the actual exposure to ECs and their effect on health.

### Conclusion

Our study supports the idea that user’s EC device adaptation, with longer inhalation times, is an ongoing process that occurs dynamically over weeks in order to maintain cotinine levels previously experienced with EC use. These compensatory vaping changes cannot be predicted based on data about demographic, clinical or surrogates of nicotine addiction. The higher inhaled volume over time is an important factor that should be considered in studies addressing the potential toxic effects of EC. These findings also reflect the importance of device adaptability when addressing public health policies regarding EC.

## Supporting information

S1 Protocol and IRB approval (PDF)

S1 TREND checklist (PDF)

S1 Table Demographic characteristics comparing subjects who failed and succeeded switching to EC during the replacement and maintenance phases. (DOCX)

S2 Table Vaping patterns of replacement phase and maintenance phase by groups. (DOCX)

S1 Fig Vaping patterns observed during the replacement phase. This graph includes all subjects that entered the replacement phase. Complete = subjects who successfully replaced tobacco smoking with electronic cigarette in a 4-week period. Early failure = subjects who could not replace tobacco smoking with electronic cigarette. Comparison between groups was statistically significant for number of vapes per day (*p = 0*.*01*), voltage *(p = 0*.*01)* and vapes-seconds (*p<0*.*001*).
(TIFF)

S2 Fig Vaping patterns observed during maintenance phase. This graph includes all subjects who entered the maintenance phase (successfully replaced TC smoking to EC vaping, N = 16). Complete = subjects who successfully continued on exclusive EC use in a 12-week period. Late failure = subjects who relapsed to TC smoking.
(TIF)
