# Supplementary material for: Differences in vaping topography in relation to adherence to exclusive electronic cigarette use in veterans
Source: PLoS One. 2018 Apr 25;13(4):e0195896. doi: 10.1371/journal.pone.0195896 (PMC5919012; doi:10.1371/journal.pone.0195896)
Supplement: S1 Protocol and IRB approval — (PDF) [file pone.0195896.s001.pdf]

Department of Veterans Affairs

Memorandum

Date: November 5, 2015

From: Associate Chief of Staff for Research (151)

Subj: Approval of Research Protocol titled "Adverse Airway Effects of Inhaled Nicotine From Tobacco and E-Cigarettes"

To: Michael Campos, M.D.

1. At its meeting of October 29, 2015, the Medical Center Research and Development Committee approved the research protocol cited above, for which you are listed as Principal Investigator. Permission to carry out this study is contingent upon your compliance with the requirements of the Research Service for the conduct of studies involving human subjects. You are approved for the participation of veteran subjects. Please be reminded that research activities conducted on VA time should only occur at VA sites, unless otherwise authorized. This project has been assigned number **#1251.09** for identification and reporting purposes.

This study was reviewed and approved by the Human Studies Subcommittee (IRB) on _10/08/2015_ and was approved for a period of _12 months_. This approval expires on _10/07/2016_.

2. The Research Office has reviewed the list of individuals who will participate in carrying out this study as provided by you on the "Report of Research Staff Members." The records in our Office for each of the following individuals are complete in that documentation of educational and training background, human studies education and appropriate certification of "Scope of Practice" meet the current Office of Research and Development (ORD) standards (Research 'Stand Down' Memorandum of March 6, 2003) and accordingly they have approval to participate in carrying out this study:

M. Campos and G. Holt

ORD has mandated that participation in a study involving human subjects of individuals who do not meet all requirements for human studies is grounds for withdrawal of approval for the study to be conducted.

If there are any changes in the educational and training background, human studies education certification or "Scope of Practice" for any of the individuals currently listed or to be added to your "Report of Research Staff Members," or if you have any questions concerning this matter, please contact the IRB Office at (305) 575-4465.

3. Since your study involves the participation of human subjects, you must be aware of and comply with the requirements for approval by the Human Studies Subcommittee (as well as by the Research and Development Committee) of the initial protocol and of any modifications. You are also responsible as Principal Investigator for obtaining informed consent and maintaining appropriate records of such consent; for maintaining a current log of patient participation and providing this log for review whenever requested; and for reporting immediately to the Pharmacy and Therapeutics Committee and to the Human Studies Subcommittee any untoward events involving human subjects that may occur in relation to your study (Adverse Drug Reaction Report).

You should already be aware of these requirements; if you have any question concerning them, please consult the Research Service Office staff. A copy of VA Form 10-1223, Report of the Subcommittee on Human Studies is enclosed for your files. While the original of this document is available in the Research Service Office, you may be asked by various groups with oversight responsibilities to provide copies and so you should have a copy in your own files.

You must provide a copy of the currently approved, date stamped Informed Consent document (VAF 10-1086) along with a copy of the Medical Center Policy Memorandum on Patients Rights and Responsibilities (MCPM 122-11-11) (see attached) to all subjects participating in this study.

In addition, you are required to have available in your study files and produce upon request the original Informed Consent document (VAF 10-1086) signed by each patient and witnessed by a person not associated with the research. You are required to hand deliver a copy of each Informed Consent document, together with a copy of the completed CPRS note entitled "T—Research Study: Documentation of Consent Discussion" as soon as possible after execution, to the IRB office (2B100). These documents will then be picked up by staff from Medical Administration Service for insertion into the patient's medical chart, both electronic and paper.

Since you have received approval for a Partial Waiver of Authorization for Recruitment Purposes for your study, you must do the following:

(1) *maintain an accurate log of each record reviewed for that research protocol.* The log must include the complete name and social security number of each subject.

4. Please note that you are required to complete a Request for Continued Approval for Human Use at an interval determined by the Human Studies Subcommittee. In addition, a Project Data Sheet must be completed for the duration of the project. You will be reminded of these requirements by the Research Office staff, and must complete the required forms by the indicated deadline. We urge you to meet this reporting responsibility in a timely fashion, since the Code of Federal Regulations require us to discontinue conduct of all research studies that do not conform. Upon completion or termination of the project, a Final Progress Report must be filed.

5. Due to recent events relating to data handling, you are being reminded that you are not authorized to remove any sensitive research-related data from the premises of this Medical Center without first obtaining the required approvals AND complying with all information that pertain to protecting confidentiality of data and patient privacy. For more information you can read guidance in the investigator's manual obtained at www.sfvafre.org or you may contact the Information Security Officer (ISO) at ext. 3361 or the Privacy Officer at ext. 7239.

6. If you have any questions concerning any of these requirements, please feel free to consult the Research Office staff.

Robert M. Jackson, M.D.

Attachments

Department of Veterans Affairs

Memorandum

Date: October 8, 2015

From: Chair, Human Studies Subcommittee
Miami VA HCS (546/151)

Subj: Approval of Partial Waiver of Authorization for Recruitment Purposes Request for Protocol: 15/125

To: Michael Campos, M.D., Principal Investigator

1. Your request for a Waiver of Authorization for Recruitment Purposes for the protocol entitled **Adverse airway effects of inhaled nicotine from tobacco and e-cigarettes** has been reviewed and approved on the date indicated above by normal review procedures pursuant to 38 CFR 16.108(d). This Waiver of Authorization has been granted under 21 CFR 164.512(i)(1)(i)(A) – "Waiver of Authorization for Uses and Disclosures for Research Purposes approved by an IRB." The approval is granted based on the determination that:
   A. the risk to the privacy of individuals is minimal based on:
      (1) the investigator's plan to protect the identifiers from improper use or disclosure
      (2) the investigator's plan to destroy the identifiers at the earliest opportunity consistent with the research and
      (3) the investigator's written assurance that the PHI identified below will not be reused or disclosed outside the VHA, except as detailed in the request for waiver of authorization
   B. the research could not practically be conducted without the waiver or alteration; and
   C. the research could not practically be conducted without access to and use of the requested information.

2. The information to be allowed to be collected under this PARTIAL Waiver of Authorization for Recruitment Purposes is limited to the following: Name, SSN, age, gender, vital status, blood and measuring urine (nicotine substance) CPRS notes, reports, drug and alcohol abuse, HIV infection, medical record number

3. If you have any questions about this approval, please contact Mitscher Gajardo, IRB Coordinator, at extention 4465.

lL

Leonardo Tamariz, M.D.

Department of Veterans Affairs | **REPORT OF SUBCOMMITTEE ON HUMAN STUDIES**

Initial Review

Project/Program Title Adverse airway effects of inhaled nicotine from tobacco and e-cigarettes

Principal Investigator Michael Campos, M.D.

VAMC Miami VAMC / 546 Review Date: October 8, 2015

COMMITTEE FINDINGS:

1. The information given in the Informed Consent under the Description of Research by Investigator is complete, accurate, and understandable to a research subject or a surrogate who possesses standard reading and comprehension skills.
   ☒ YES ☐ NO

2. The informed consent is obtained by the principal investigator or a trained and supervised designate under suitable circumstances.
   ☒ YES ☐ NO

3. Every effort has been made to decrease risk to subject(s)?
   ☒ YES ☐ NO

4. The potential research benefits justify the risk to subject(s)?
   ☒ YES ☐ NO

5. If subject is incompetent and surrogate consent is obtained, have all of the following conditions been met; a) the research can't be done on competent subjects; b) there is no risk to the subject, or if risk exists the direct benefit to subject is substantially greater; c) if an incompetent subject resists, he will not have to participate; d) if there exists any question about the subject's competency, the basis for decision on competency has been fully described.
   ☒ YES ☐ NO

6. If the subject is paid the payment is reasonable and commensurate with the subject's contribution.
   ☒ YES ☐ NO ☐ NA

7. Members of minority groups and women have been included in the study population whenever possible and scientifically desirable.
   ☒ YES ☐ NO

8. Comments: (Indicate if Expedited Review) Initial Review

This study was approved for 364 day from the review date indicated above. A Partial Waiver of Authorization for Recruitment Purposes has been approved for this study (see attached approval letter)

☒ APPROVE ☐ DISAPPROVE/REVISE

SIGNATURE OF CHAIRMAN
Leonardo Tamariz, M.D., Chairperson

DATE
IRB Approval: 10/22/2015
Expiration: 10/07/2016

VA Form 10-1223
OCT 1995(R)

Department of Veterans Affairs

Memorandum

Date: June 7, 2015

From: Principal Investigator: Campos, Michael MD

Subj: Location of Research Project/ Research Sites

To: Chair, Biosafety Subcommittee

Title of Study: Evaluation Of Acute And Subacute Effects Of Nicotine Electronic Cigarette Vapors

For research within the VA, please indicate location and/or room numbers where study activities will occur:<br>VA Medical Center in Miami division of Pulmonology who's offices are located on the eighth floor in rooms B821, B822, B823, A807, A808, A809, A810

1) **Research Sites** *(check all that apply)*:

- ☒ **Miami VA HCS facilities** *(list)*:
- ☐ **University of Miami Hospital and Clinics**
- ☒ **University of Miami Medical Campus (Bascom Palmer, Sylvester, Rosentiel Building, Luis Pope etc...)**
- ☐ **Nova University**
- ☐ **Florida International University**
- ☐ **Other Florida University** *(list)*:
- ☐ **Miami Jewish Home and Hospital for the Aged**
- ☐ **International study** *(list the countries)*:
- ☐ **Other cooperative research or multi-center trial where the PI is the lead investigator**
- ☐ **Other** *(list)*:

Note that substantial change in the site where the research will be conducted requires a revision of the protocol and/or biosafety and chemical safety plans describing the new location. (This change must be submitted as an amendment to the protocol.)

Signatures acknowledge approval of location and space utilization:

Principal Investigator: MICHAEL CAMPOS ______ [signature]
Please print — Signature

Automated VA FORM 2105

August 24, 2006

ADVERSE AIRWAY EFFECTS OF INHALED NICOTINE FROM TOBACCO AND E-CIGARETTES

The current protocol is part of a larger collaborator study between the Miami VA Medical Center and the University of Miami aimed at evaluating the toxicity of electronic cigarettes (ECs). This sub-study will specifically enroll active smokers at the Miami VA to compare the effects of switching from active smoking to e-cigs versus continuation of smoking. As a secondary outcome, we will evaluate the feasibility of using EC as a tool for weaning from tobacco use.

BACKGROUND AND SIGNIFICANCE

Importance of the problem: Smoking has innumerous adverse effects on human health. From a respiratory point of view, chronic bronchitis and chronic obstructive pulmonary disease (COPD) in the US and Florida are predominantly due to either active or passive chronic tobacco smoke exposure. The pathogenesis of these conditions involves the induction of smoke-mediated inflammation, shown to be in part due to increased TGF-β signaling (1-4). The recent introduction of electronic cigarettes (ECs) to provide habitual smokers a source of nicotine without the need to inhale tobacco smoke is thought to reduce toxicity to airway epithelial cells. The use of ECs decreases use of traditional cigarettes (5), diminishes nicotine withdrawal symptoms (6, 7), leads to opposite effects on exhaled NO levels compared to tobacco (8, 9), and may diminish stimulation of reward centers in the brain leading to less dependence due to the lack of other tobacco-derived chemicals (10). While this may be beneficial, the medical community's concerns over unregulated EC use leading to increased nicotine addiction (11, 12) appears warranted given the rapid rise in popularity amongst teenagers (13). The manufacturers of ECs tout comparative safety as a reason to switch from traditional tobacco cigarettes to their products. However the dearth of data supporting this claim led the FDA to issue warning letters to these companies (14).

For now, the World Health Organization recommends against EC use (15) until ECs have been properly evaluated. On the other hand, the American Association of Public Health Physicians (16) support the use of ECs as a means of "Harms Reduction" (17, 18) since ECs provide nicotine without the myriad of other toxic chemicals produced from burning tobacco. These recommendations are mostly due to theoretical considerations and expert opinion. Due to the novelty of these devices, many concerns of their safety on health and long-term nicotine addiction remain unanswered due to a lack of studies that comprehensively evaluate their toxicity.

Current studies on EC vapors are conflicting and reveal that they produce either similar (19-21) or reduced (6, 22) effects on systemic nicotine levels, carcinogen levels and cardiovascular effects compared to other nicotine replacement strategies. Side effects of ECs include mouth/throat irritation (23), vertigo (23), headaches (23), bronchospasm (8, 24) and nausea (25) similar to those found with approved nicotine replacement therapies (NRT).

ECs produce nicotine vapors of a particle size that deposit the vapor in the lower respiratory tract (26, 27), unlike the larger sized particles of approved inhalation NRT devices that remain in the oropharynx (28, 29). This fact may explain why EC vaping but not NRT use has been associated with lipoid pneumonia (30) and bronchospasm (8, 24). It raises concerns that the toxicity of components of EC such as diluents may have significant health risks (31), in addition to the known risks of nicotine alone (32). The lack of consistent results demands a full evaluation of EC toxicity.

In 2009, the FDA reported that a cartridge of an electronic cigarette contained diethylene glycol (33), even though other studies failed to identify this substance. The FDA testing also identified

tobacco-specific nitrosamines in ECs (33). Both substances are known carcinogens. However, they can also be found in nicotine gums and nicotine inhalers (34). Other harmful substances were found as well, including acrolein (35). The French national consumer agency analyzed the vapor of ECs and found formaldehyde levels matching those from tobacco cigarettes (36). On the other hand, the test conditions did not necessarily mimic real life scenarios. Therefore, exposure systems mimicking real life situations with an appropriate evaluation of toxicity are desperately needed, especially since the use of ECs has increased dramatically in recent years. Our proposal is designed to answer this question by investigating the safety of ECs in primary cultures of normal human bronchial epithelial (NHBE) cells and in clinical trials with individuals who are non-smokers or who replace tobacco smoking with EC vaping.

ECs produce vapor mainly from propylene glycol (PG) or vegetable glycerin (VG) that are blended at different concentrations, making comprehensive testing more difficult, especially when considering additional flavoring (which will not be addressed in this proposal). There are three major types of ECs on the market:

- Mini (cig-a-like), available with 0, 6, 12, 18 and 36 mg/ml of nicotine.
- Mid-size (vapor pens), available with 0, 6, 12, 18, 24 and 36 mg/ml of nicotine.
- Advanced personal vaporizers (APV) or MODS that deliver more vapor per puff using the same e-liquid and nicotine concentrations.

Since the major difference between these devices is the amount of vapor produced per puff, in this sub study we will focus on a commonly used mid-sized EC: eVic® (Joyetech Co., Ltd., ShenZhen China) with variable nicotine concentrations depending on patient use.

EXPERIMENTAL APPROACH

The study will evaluate the effects of inhaling EC vapor containing nicotine when used as a tobacco replacement tool. **By using active cigarette smokers as a comparator group, the study will increase our knowledge of whether the use of ECs is more or less harmful than continuation of tobacco smoking**. This study will examine the *in vivo* effects of EC vapors in nasal ion transport and TGF-β levels as well as in serum inflammatory biomarkers.

As the primary endpoint, nasal ion transport will be assessed by nasal potential difference (NPD), which measures the voltage potential resulting from epithelial ion fluxes (both Na+ absorption and Cl- and K+ secretion, see 37, 38) at the mucosal surface *in vivo*. In normal airway epithelia, Na^+ absorption is the primary ion transport activity so that the resulting airway surface potential difference is negative with reference to the interstitium (39). Ion transport across nasal epithelia is representative for findings in distal airways (40, 41).

We also believe that NPD is a reasonably sensitive and minimally invasive biomarker for this study as discussed below. We will use NPD as a primary endpoint because changes in most clinical parameters will only be seen after months or even years of exposure. For that particular reason, there is a recent surge to find alternative biomarkers that may be used as surrogate endpoints in shorter clinical trials. Since NPD directly measures the changes in ion transport expected to influence mucociliary clearance (MCC) and therefore overall outcome and since changes of NPD measurements are indirectly linked to lung function changes and MCC in trials with cystic fibrosis patients (44, 45), we believe that NPD lends itself as a reasonable surrogate for MCC for this clinical trial.

As secondary endpoints, we will also measure TGF-ß levels in nasal secretions and expression in nasal cells to correlate these with the level of CFTR and CaCC-mediated Cl^- conductance (CFTR is known to show decreased function with elevated TGF-β) (42, 43). In addition plasma

samples will be collected to study systemic markers of inflammation as well as toxic substances that may result from vaping and smoking

EXPERIMENTAL DESIGN

The design is a smoking replacement trial, in which we will compare changes in upper airway ion transport and inflammation in smokers who switch to EC vaping versus subjects that continue to smoke tobacco cigarettes. In addition, we will use the trial design to evaluate the feasibility of using the EC as a means to quit tobacco use.

Study population/recruitment. We plan to study the effects of continuous EC vaping (3 months) in 30 active smokers that successfully switch to ECs (a smoking replacement trial). For this, we plan to enroll approximately 200 active smokers (screening phase), expecting that only one fourth will be able to stay abstinent from tobacco and keep exclusively vaping the ECs after a month. The 30 subjects who can remain tobacco-smoke free for the initial 4 weeks will undergo the study procedures, as >90% of them are likely to remain on ECs for the next few months (49) (as opposed to what is observed in a smoking cessation trial (48)). Twenty additional smokers who wish to continue to smoke will be enrolled as well for the comparator group. Subjects that actively smoke and have a history of at least 5 pack-years will be invited to participate. Exclusion criteria include HIV, marijuana use, COPD, asthma and other concomitant inflammatory pulmonary disorders, subjects with known pulmonary diseases including malignancies, subjects with prior thoracic surgery and subjects that used oral corticosteroids or antibiotics within the prior 3 months, subjects with allergies to study medications and subjects incapable of providing informed consent. Subjects must be in stable condition.

Subjects will be enrolled primarily in the pulmonary function test laboratory, pulmonary and primary care clinics. We will place IRB-approved advertisements in clinics if necessary. Recruitment will occur over a course of 5 years.

The study will have 4 Phases: transition, stability, weaning and surveillance.

Phase I: transition from tobacco use to EC (4 weeks): After signing informed consent, all subjects entering the screening phase will have pulmonary function testing (PFT) if none available in the prior month and we will record basic demographic and social history. After obtaining an initial blood sample (plasma), we will provide and instruct the subjects how to use the EC (eVic®). The nicotine content of the "e-liquid" provided (a PG:VG mix 50:50) will match the number of cigarettes smoked a day and will vary from subject to subject (see below for calculation). We will use the 12 mg/ml concentration e-liquid. During this "transition phase" period we will assess their success of abstaining from smoking tobacco. To monitor abstinence to tobacco smoking, subjects will be seen in person weekly for measurements of exhaled CO (ExCO), and venous COHb. ExCO > 6 ppm (50) or venous COHb >1.6% (51) will be considered the result of tobacco cigarette use. In addition, we will measure urinary marijuana with a qualitative test and if positive we will exclude the subject from the study. During those visits we will assess vaping compliance by downloading the vaping history of the EC using the myVapors® software, available from the EC manufacturer and will collect data regarding potential side effects. Subjects will be encouraged to transition solely to EC within the first 4 weeks. Blood draws for biomarker assessment will be performed in each visit of this phase.

Phase II: Stability on EC (12 weeks): In this phase, subjects will remain on EC use for three months. In this phase, all subjects will continue to use ECs at a stable nicotine concentration for additional 12 weeks. Tobacco use abstinence will be monitored every week as in the transition phase as detailed in Table 1. High ExCO levels will be confirmed with venous COHb if necessary. Oral washes and nasal lavages will be done at the beginning and the end of this phase.

Smoker controls: 20 tobacco smokers will be directly enrolled in the “stability” phase, where they will continue to use their regular tobacco for 3 months. They will undergo nasal samplings and NPDs before and after, with monthly blood collections. After this phase, these control subjects may be re-enrolled in the study to start in the transition phase with EC.

Inhaled nicotine dose calculation: We will match the subjects’ daily nicotine dose when switching from tobacco smoking to EC vaping. A Kentucky 3R4F research cigarette contains ~0.7 mg nicotine (http://www2.ca.uky.edu/refcig/3R4F%20Preliminary%20Analysis.pdf) and is consumed in 9 puffs. The equivalent of 1 pack per day (180 puffs, 14 mg of nicotine) would be an eVic® 1.2 ml cartridge containing 12 mg/ml of nicotine (cartridges are available as 6, 12, 18, 24, 36 and 48 mg/ml). One cartridge provides 180-200 puffs. Given that nicotine content in “e-liquids” is variable, we expect subjects to adjust their number of puffs or cartridges a day according to their needs or preferences. Since the EC chosen will count the puffs, we will have a record of use.

Statistical Analysis: An n = 50, 30 EC users and 20 smokers, gives us a power of >98% for detecting a difference in TGF-β levels. We will analyze NPD parameters as described above. Results will be adjusted to nicotine exposure (urinary nicotine and cotinine levels) and inhalation (EC puffs per day).

Experimental Methods

PROCEDURES PERFORMED AT THE MIAMI VAMC

Adverse Event recording: We will record information about respiratory symptoms and other potential adverse events. Patients will also receive weekly calls to gather information about tobacco smoking abstinence, symptoms and potential adverse events.

Pulmonary function testing (PFT) will be performed according the American Thoracic Society (ATS) recommendations (52). Participants will be asked to refrain from using short-acting bronchodilators for at least 4 hours before testing if applicable. Lung volumes and D_LCO will be performed following ATS recommendations (53, 54). Height, weight and oxygen saturation will be measured prior to these tests.

Carboxyhemoglobin (COHb) will be measured in venous blood samples by co-oximetry (pHOx®ULtra, Nova Biomedical, Waltham, MA). Exhaled Carbon Monoxide (ExCO) will be measured by the handheld Smokerlyzer™ device (Covita, UK).

Collection of plasma: Blood samples will be collected in lavender-top tubes and transported to the Pulmonary Research Laboratory at the Miami VA. Plasma will be collected using a standard protocol. A full 8.5 mL of blood will be collected. This will produce about 2.5-3 mL of plasma. Tubes will be centrifuged at 2000-3000 RCF at 4°C for 15 min. Plasma will then be transferred in 1-mL aliquots to pre-labeled cryovials and freezed at –80°C until used. We will use to measure biomarkers and cotinine levels.

Biomarkers: We will measure relevant inflammatory and toxic metabolites in EBC and plasma, including H_2O_2, 8-isoprostane, IL-8, C-reactive protein, nicotine metabolites, coagulation markers, thiocyanate, acrolein. Subject’s consent will be obtained from participants to store residual samples after analysis for future studies.

Oral washes: Oral washes will be performed by having participants gargle with 10 ml sterile 0.9% saline. These washes will be centrifuged and stored at -80°C for future studies (i.e microbiome).

Leukosorb collection of nasal fluid: After 100 µL normal saline will have been sprayed into the nose, a small paper will be inserted and the nostril closed with a nose clip for 2 minutes. Then, the paper will be retracted and eluted for cytokine analysis in the laboratory (56). No adverse effects are known.

PROCEDURES PERFORMED AT THE UNIVERSITY OF MIAMI

Nasal potential difference (NPD) measurements: This will be our primary outcome. Since tobacco smoke changes NPD, we will assess whether EC vapors with nicotine elicit a similar effect. Subjects will be instructed to exhale through the nose when vaping. NPD will be measured as previously described (47) and as detailed in Human Subjects. Effects on CFTR and CaCC-mediated ion transport will be evaluated. We will also collect nasal cells and lavages to measure TGF-ß levels (mRNA and protein by ELISA) and correlate these with the level of CFTR- and CaCC-mediated Cl^- conductance.

Nasal lavages will be performed using 10cc of normal saline will be harvested to measure inflammatory markers.

Nasal brushings will be performed to harvest nasal epithelial cells for assessment of mRNA changes (cytokines and other inflammatory molecules).

Exhaled NO measurements (breath and nasal) will be performed as a marker of inflammation.

Plasma Cotinine levels measurements 200ul of plasma of each study subject will be send to the **University of Miami Rosentiel Medical Science Building; Pulmonary Research Department Room # 7052.**

Leukosorb collection of nasal fluid: After 100 µL normal saline will have been sprayed into the nose, a small paper will be inserted and the nostril closed with a nose clip for 2 minutes. Then, the paper will be retracted and eluted for cytokine analysis in the laboratory (56). No adverse effects are known.

The following **Table 1** outlines the timing of the above procedures in relation to study visits.

		TRANSITION PHASE				STABILITY PHASE											
Time (weeks)	E	1	2	3	4	5	6	7	8	9	10	11	12	13	14	15	16
PROCEDURE	enrollment	V1	V2	V3	V4	V5	V6	V7	V8	V9	V10	V11	V12	V13	V14	V15	V16
ICF	X																
baseline questionnaire	X																
FU smoking questionnaire	X	X	X	X	X	X		X		X		X		X		X	X
e-liquid refill	X	X	X	X	X	X	X	X	X	X	X	X	X	X	X	X	X
Ex CO	X	X	X	X	X	X	X	X	X	X	X	X	X	X	X	X	X
venous COHb	X	X	X	X	X	X			X				X				X
marihuana screen	X				X												X
NPD at UM						X											X
nasal lavage						X											X
nasal brush						X											X
PFT	X																
Spirometry only																	X
Mouth wash	X								X								X
plasma	X	X	X	X	X	X			X				X				X

time (weeks)	E	1	2	3	4	5	6	7	8	9	10	11	12	13	14	15	16
PAYMENTS	enrollment	V1	V2	V3	V4	V5 baseline NPD at UM	V6	V7	V8	V9	V10	V11	V12	V13	V14	V15	V16 post NPD at UM
	$0.00	$0.00	$0.00	$0.00	$0.00	**$100.00**	$0.00	$0.00	$0.00	$0.00	$0.00	$0.00	$0.00	$0.00	$0.00	$0.00	**$200.00**

Payment to participants: Participants who complete the first 4 visits (screening phase) and qualify for first nasal sampling and NPD measurement, will receive a payment of $100.00. After this they will enter a 12-week phase of consistent use of either EC or tobacco smoking and receive a payment of $200.00 after completing the final visit with nasal sampling and second NPD measurement. Compensation will not be provided at every visit but rather after completion of each

study phase to enhace the chances that the subject will succesfully quit tobacco cigarettes and switch to EC. It's well known that smoking cessation is difficult and that reward mechanisms increase quitting rates. Although the payments are not "rewards", the payment schedule can be used as a motivation to quit.

Planned enrollment: The study will be performed throughout a 5-year period. Recruitment of smokers unwilling to quit and smokers that will attempt to switch to EC will be recruited as follows:

PLANNED ENROLLMENT	YEAR 1				
	Jul - 2015 - Dec		Jan - 2016 - Jun		TOTAL Y1
	Q1	Q2	Q3	Q4	
SMOKERS NO E-CIGS	0	2	2	2	6
SMOKERS NPD1	0	2	2	2	6
SMOKERS NPD2	0	0	2	2	4
POTENTIAL E-CIG USERS	4	4	8	4	20
E-CIG NPD1*	1	1	2	1	5
E-CIG NPD2	0	1	1	2	4

* assuming a 25% e-cig retention

	YEAR 2				
	Jul - 2016 - Dec		Jan - 2017 - Jun		TOTAL Y2
	Q1	Q2	Q3	Q4	
SMOKERS NO E-CIGS	0	0	2	2	4
SMOKERS NPD1	0	0	2	2	4
SMOKERS NPD2	2	0	0	2	4
POTENTIAL E-CIG USERS	4	4	4	4	16
E-CIG NPD1*	1	1	1	1	4
E-CIG NPD2	1	1	1	1	4

* assuming a 25% e-cig retention

	YEAR 3				
	Jul - 2017 - Dec		Jan - 2018 - Jun		TOTAL Y3
	Q1	Q2	Q3	Q4	
SMOKERS NO E-CIGS	0	0	2	2	4
SMOKERS NPD1	0	0	2	2	4
SMOKERS NPD2	2	0	0	2	4
POTENTIAL E-CIG USERS	8	8	8	4	28
E-CIG NPD1*	2	2	2	1	7
E-CIG NPD2	1	2	2	2	7

* assuming a 25% e-cig retention

	YEAR 4				
	Jul - 2018 - Dec		Jan - 2019 - Jun		TOTAL Y4
	Q1	Q2	Q3	Q4	
SMOKERS NO E-CIGS	0	0	0	2	2
SMOKERS NPD1	0	0	0	2	2
SMOKERS NPD2	2	0	0	0	2
POTENTIAL E-CIG USERS	8	8	8	8	32
E-CIG NPD1*	2	2	2	2	8
E-CIG NPD2	1	2	2	2	7

* assuming a 25% e-cig retention

	YEAR 5					
	Jul - 2019 - Dec		Jan - 2020 - Jun		TOTAL Y5	
	Q1	Q2	Q3	Q4		STUDY TOTAL
SMOKERS NO E-CIGS	0	2	2	0	4	20
SMOKERS NPD1	0	2	2	0	4	20
SMOKERS NPD2	2	0	2	2	6	20
POTENTIAL E-CIG USERS	8	8	8	0	24	120
E-CIG NPD1*	2	2	2	0	6	30
E-CIG NPD2	2	2	2	2	8	30

* assuming a 25% e-cig retention

EXPECTED RESULTS, POTENTIAL DIFFICULTIES, AND ALTERNATIVE STRATEGIES

The importance of this study design is that it mimics real world use of ECs. These devices are being widely promoted as an alternative form of smoking cessation with many switching to but not weaning from them. This is in part because of the unproven statement that tobacco-free products are safer and their indoor use is unregulated, which theoretically allows an increased nicotine exposure. For the trial, we chose to study the most commonly used EC type and the most frequently used e-liquid mix of PG and VG (50:50).

The effects of EC on airway TGF-β and ion exchange are hard to predict at this point. We expect that the presence of nicotine will significantly affect NPD similar to what regular tobacco cigarettes do. If the PG:VG mix has no local toxic effects in this subacute exposure (determined in other aspects of the study), it is possible that TGF-β levels will improve as the duration of tobacco cigarette abstinence increases (nasal samples), but nicotine could maintain these levels seen with smoking. If this is the case, it will provide valuable information that replacing regular cigarettes with EC is not less toxic or harmful as is generally perceived.

The initial phase of screening more smokers than required to complete the analysis is necessary to avoid performing semi-invasive and time consuming procedures such as NPD in subjects likely to relapse back to tobacco use, as well as to avoid the phase of dysregulated airway inflammation that may occur when smoking cessation occurs (55). Although the subjects undergoing study procedures will do so after 4 weeks of already using ECs, we expect to still detect significant differences in the data collected 3 months later if there is cumulative toxicity in the long term.

Although we do not anticipate major problems in subject recruitment at the VA, in case of slow enrollment, we will extend study invitations to the 10 clinic-base outpatient centers that are part of the Miami VA health system. These clinics nearly double the Miami VA population to close to 50,000 subjects. We do not expect complications executing any of the study procedures. Assurance of tobacco cessation will be monitored objectively every 2 weeks in different types of samples (exhaled air and blood) to maximize detection of non-compliance. Although it is possible that some subjects may elude these surveillance tests at a certain time, it is unlikely that this may occur for every checkpoint. If there is suspicion that a subject may be smoking, based on interview or equivocal test results, we will opt to exclude the subject from the study and continue expanding enrollment. If dropout in the EC use arm is higher than anticipated, we will recruit more subjects to reach the final number of 30 completing the study on ECs. This is unlikely since there is a very high adherence to ECs in a nicotine replacement environment for the relatively short total duration of 3 month (49).

HUMAN SUBJECTS

THE STUDY MEETS THE DEFINITION OF HUMAN SUBJECTS RESEARCH AND THE DEFINITION OF A 'CLINICAL TRIAL'. WILL BE REGISTERED ON WWW.CLINICALTRIALS.GOV)

1. Risks to the Subjects

A) Human Subjects Involvement and Characteristics: We will enroll active smokers older than 18 years, who actively smoke and have a smoking history of at least 5 pack-years. These subjects should not be currently using ECs but should be willing to replace tobacco cigarettes to ECs. All subjects will undergo lung function testing to exclude chronic obstructive pulmonary disease, as this condition may not only increase the risk of procedures but also impose additional inflammatory confounders. Exclusion criteria include the presence of airflow obstruction (FEV1/FVC < LLN), other concomitant inflammatory pulmonary disorders (i.e asthma), subjects with known pulmonary malignancies, subjects with prior thoracic surgery and subjects that used oral corticosteroids or antibiotics within the prior 3 months, subjects with allergies to study medications and subjects incapable of providing informed consent. Subjects must be in stable condition. Considering that about 75% will not be able to stay abstinent from tobacco cigarette smoking, we expect to screen 120 subjects, who meet these criteria, to be able to have 30 subjects who will complete all study procedures 3 months apart. In addition, we will enroll 20 active smokers unwilling to quit tobacco smoking but willing to undergo the study procedures using the same exclusion criteria. Since these trials will be performed at the Miami Veterans Affairs (VA) Medical Center, we expect that subjects will be >90% men, 20% Hispanic and 25% African American.

B) Sources of Materials: The following are the procedures that will be performed:

Collection of nasal lavage fluid: Nasal lavage fluid will be used to measure changes in epithelial inflammatory cytokines. The lavage will be performed using saline pre-warmed to 37˚C. The subject will keep the head bent forward with the face held horizontally, while the left nasal cavity is filled with saline, using a 10 ml syringe connected to the nostril via a short tube and a nasal olive. After 5 minutes approximately 5 ml of the saline is recovered by aspiration. The samples will be centrifuged to remove cellular debris and aliquots of the supernatants stored at - 20°C in microcentrifuge tubes until analysis.

Harvesting of nasal epithelial cells will be performed following the nasal lavage to obtain nasal epithelial cells for the assessment of nasal epithelial cytokine mRNA expression. For this, nasal mucosal cells will be harvested from the right nasal cavity by a gentle nasal brushing using a 5.5 mm diameter nylon brush (Doft AB, Östhammar, Sweden). The brush will be immediately placed in a tube containing physiological saline and twirled for 3-5 seconds. Cells will be centrifuged and the pellet re-suspended in RNA*later*® and stored at -80˚C for later analysis.

Nasal Potential Difference (NPD). Clean, double lumen nasal catheters with an external diameter of 2.5 mm will be used for measurements. One lumen will be filled with freshly prepared agar; the other lumen will be used for perfusion with different solutions. The agar catheter and subcutaneous bridges (metal butterfly needle system) will be made immediately prior to use by warming 3% agar gel and injecting the solution into the catheter. They will be attached to 3M KCl/calomel reference electrodes (Baxter; Deerfield, IL) and connected to a bioamplifier system (AD Instruments; Colorado Springs, CO) for measuring and recording. The catheter tip will be fixed at the location of the most negative potential in the area under the inferior meatus. The test will be initiated after a stable value (± 0.5 mV over 30 s) is obtained using perfusion with Ringer solutions. Then, the nose will be rinsed with different solutions containing amiloride (to block ENaC), no chloride (increase CFTR conductance), isoproterenol (activate CFTR) and ATP (stimulate CaCC). In the other nostril, the same experiment will be repeated, but we will attempt to maximize the CaCC signal. For this, we will initially also block ENaC with amiloride, and

equilibrate the chloride concentrations with perfusion of the nasal epithelium with chloride free solution. From there, we will not stimulate the CFTR with isoproterenol, but will directly use ATP to increase the intracellular calcium concentration and activate CaCC. The rinsing with chloride free solution is necessary to not confuse passive chloride flow through CFTR based on gradient differences with flow through ATP activated CaCC.

Blood samples will be obtained to measure venous COHb, inflammatory markers and toxic metabolites. 2 standard EDTA 5 ml tubes will be collected to harvest plasma. A 1 ml heparinized syringe will be used for the venous COHb.

Pulmonary Function Testing will be performed according to American Thoracic Society recommendations if not done within 1 month prior to enrollment. Participants will be asked to refrain from using short-acting bronchodilator drugs for at least 4 hours before testing. Lung volumes and D_LCO will also be performed following ATS recommendations. Height, weight and oxygen saturation will be measured prior to these tests.

C) Potential Risks

Collection of nasal lavage fluid and harvesting of nasal epithelial cells: this procedure carries minimal risks to the subject. It is sometimes associated with an unpleasant feeling due to the lavage, minimal risk of aspiration of part the 5 ml of sterile saline solution, and coughing. To prevent this from happening, the head of the patient will be bend forward during the collection, so the fluid will rinse out of the nose and not get into the pharynx where it can be swallowed or aspirated. Cell sampling with the microbrush can cause a mild discomfort and a mild nosebleed.

NPD measurement is a non-invasive method with minor risks, which includes swallowing and aspiration of the solution used for rinsing the nose. Even in case of accidental swallowing or aspiration, there are limited risks of a severe reaction, even in a respiratory impaired individual. Amiloride is a diuretic medication and can increase urine production when given in a higher dose, which is not expected here as the used dose is low and systemic absorption is not anticipated. Isoproterenol is a beta mimetic and can lead to tachycardia, hypertension and migraine. The dose used is low and the systemic absorption negligible. Other potential side effects include nosebleed, runny nose and coughing. For the study, a subcutaneous insertion of a butterfly needle is required, which brings a minimal risk of bruising, bleeding and infection. The butterfly is only used as a control electrode. No medications are injected through it into the skin or vascular system.

Collection of clinical information (demographics, adverse events): No significant physical risks arise from these procedures. There is always the risk of psychological distress and breach of confidentiality. In order to minimize this risk, written information is stored in locked files or file-rooms when not attended by study personnel. Data will be transformed to an electronic database using REDCap (registered under the University of Miami).

Blood draw: The risks from blood draw from a vein are minimal but include discomfort at the site of puncture, possible bruising around the puncture site, rarely an infection, and uncommonly, faintness from the procedure.

Pulmonary Function Testing is a routine clinical procedure with few risks. Patients are coached to make repeated forceful breathing efforts. The subjects might have chest soreness. Unusually, subjects may become lightheaded during these efforts. We will minimize this risk by having the PFT done in a sitting position.

EBC collection: may produce dizziness if the subject hyperventilates. We will minimize this risk by coaching subjects to keep a slow and paced respiration.

Electronic cigarettes: Known side effects of ECs include mouth/throat irritation (23), vertigo (23), headaches (23), bronchospasm (8, 24), and nausea (25) similar to those found with approved

nicotine replacement therapies (NRT). Other potential toxic effects are unknown and the goal of this proposal.

2. Adequacy of Protection Against Risks

A) Recruitment and Informed Consent: Active smokers (N=140) will be recruited at the Miami VA Medical Center pulmonary function test laboratory as well as pulmonary and primary care clinics. IRB-approved advertisement and flyers will be used if necessary. We may also contact directly health care providers to refer active smokers.

Study candidates will be briefed about the study protocol and procedures and if interested will be invited to come on a separate day, where study investigators will explain the study objectives, process risks and benefits. Subjects will take the informed consent home and come on a follow-up day to sign and be enrolled. Subjects will have sufficient time to meet with the PI to address all their questions about the research.

Informed consent: The study protocols and their informed consent documents will be submitted for review to the Miami VA IRB. Subjects interested in volunteering for the study will have a lengthy discussion with the investigators about the risks and benefits of their participation.

B) Protection Against Risk:

General: We will carefully select subjects that can tolerate the procedures of the study. Our Human Research Laboratories and Pulmonary Function Laboratory are fully equipped to handle possible fainting and other, more serious types of medical emergencies. A CPR cart will be available, as will adequate personnel (physicians trained in BLS and ACLS) to handle any potential emergency.

Clinical and personal information: All samples will be labeled with unique codes to protect the subject's identity. Samples will be catalogued using Freezerworks® software. There will be no way to match samples to subjects. All patient data will be stored in locked file cabinets or a room that is locked when unattended. All electronic data will be secured using password protection. Access to these data will be restricted to the research staff only. The study investigators will regularly monitor for safety issues.

Electronic cigarettes: At the present time there are no health warnings regarding the use of ECs based on objective data of their toxicity. The health warning is a word of caution due to the lack of these studies. Given that assessment of EC toxicity is the main goal of this proposal, subjects will be informed that risks are not well established and that they need to report any potential side effects immediately. It is possible that subjects may be allergic to components of the e-liquids (PG is more commonly mentioned in online blogs) with mild symptoms such as hoarseness and itchy throat. If any of these potential symptoms occur, subjects will have a complete clinical evaluation including additional lung function testing. The investigators will then consider withdrawal of the study depending on severity.

NPD: Having the patient bending the head forward will minimize the risk of aspiration of nasal irrigation solutions, by allowing the solution to drip out of the nose and not in the pharynx. The perfusion rate will be set at 0.5 ml/min for the nose to be rinsed with very low volumes.

Pulmonary Function Testing: Unusually, subjects may become lightheaded during these efforts. We will minimize this risk by having the PFT done in a sitting position.

3. Potential Benefits of the Proposed Research to the Subjects and Others

Subjects will not receive any direct benefit from participating in this project.

4. Importance of the Knowledge to Be Gained

The experiments to be done with the samples obtained from these subjects aim at understanding the potential toxicity of electronic cigarettes, devices of growing popularity that are currently unregulated. The knowledge gained may lead to potential new regulations about the use of these devices in the future.

5. Data Safety and Monitoring Plan

The study investigators will provide ongoing review of adverse event data. They will discuss adverse events with a committee formed by additional faculty members within the Pulmonary Division that can stop the study. Additional oversight will be provided by the IRB to whom adverse events and serious adverse events will be reported.

The study physicians and research coordinator will continually monitor the well being of all patients enrolled in the trial. The project members will meet monthly to review accruals to protocols, accrual goals, responses to protocol treatments, and serious adverse events. For individual side effects, study participation (and use of EC) will be discontinued. For group side effects, protocol modifications will be addressed accordingly and submitted to the IRB before they are implemented. Found toxicity will be reported to the FDA as well. No modifications to protocols are implemented until they are approved by the IRB. If changes are required to the consent form as a result of the serious adverse events, the consent is revised and submitted to the IRB for review and approval. All subjects are re-consented when the new information is being provided to and/or new risks are identified.

Inclusion of Women and Minorities

We have no exclusion criteria regarding gender or ethnicity.

Inclusion of Children

Although possible, the inclusion criteria of having at least 5 years of smoking makes it unlikely that children (aged between 18 and 21) will be enrolled.

Bibliography & References Cited

1. Ning W, Li CJ, Kaminski N, Feghali-Bostwick CA, Alber SM, Di YP, Otterbein SL, Song R, Hayashi S, Zhou Z, Pinsky DJ, Watkins SC, Pilewski JM, Sciurba FC, Peters DG, Hogg JC, Choi AM. Comprehensive gene expression profiles reveal pathways related to the pathogenesis of chronic obstructive pulmonary disease. *Proc Natl Acad Sci U S A* 2004;101:14895-14900.
2. Ogawa E, Elliott WM, Hughes F, Eichholtz TJ, Hogg JC, Hayashi S. Latent adenoviral infection induces production of growth factors relevant to airway remodeling in copd. *Am J Physiol Lung Cell Mol Physiol* 2004;286:L189-197.
3. Takizawa H, Tanaka M, Takami K, Ohtoshi T, Ito K, Satoh M, Okada Y, Yamasawa F, Nakahara K, Umeda A. Increased expression of transforming growth factor-beta1 in small airway epithelium from tobacco smokers and patients with chronic obstructive pulmonary disease (copd). *Am J Respir Crit Care Med* 2001;163:1476-1483.
4. Zou W, Zou Y, Zhao Z, Li B, Ran P. Nicotine-induced epithelial-mesenchymal transition via wnt/beta-catenin signaling in human airway epithelial cells. *Am J Physiol Lung Cell Mol Physiol* 2013;304:L199-209.
5. Polosa R, Morjaria JB, Caponnetto P, Campagna D, Russo C, Alamo A, Amaradio M, Fisichella A. Effectiveness and tolerability of electronic cigarette in real-life: A 24-month prospective observational study. *Intern Emerg Med* 2013.
6. Laugesen M. Safety report on the ruyan® e-cigarette cartridge and inhaled aerosol. 2008. Available from: http://www.healthnz.co.nz/RuyanCartridgeReport30-Oct-08.pdf.
7. Dawkins L, Turner J, Hasna S, Soar K. The electronic-cigarette: Effects on desire to smoke, withdrawal symptoms and cognition. *Addict Behav* 2012;37:970-973.
8. Vardavas CI, Anagnostopoulos N, Kougias M, Evangelopoulou V, Connolly GN, Behrakis PK. Short-term pulmonary effects of using an electronic cigarette: Impact on respiratory flow resistance, impedance, and exhaled nitric oxide. *Chest* 2012;141:1400-1406.
9. Chambers DC, Tunnicliffe WS, Ayres JG. Acute inhalation of cigarette smoke increases lower respiratory tract nitric oxide concentrations. *Thorax* 1998;53:677-679.
10. Lewis A, Miller JH, Lea RA. Monoamine oxidase and tobacco dependence. *Neurotoxicology* 2007;28:182-195.
11. U.S. FDA. Electronic cigarettes. 2013. Available from: http://www.fda.gov/newsevents/publichealthfocus/ucm172906.htm.
12. Council on Science and Public Health AMA. Use of electronic cigarettes in smoking cessation programs. 2013. Available from: http://www.ama-assn.org//resources/doc/csaph/a10csaph6ft.pdf.
13. Center for Disease Control and Prevention. E-cigarette use more than doubles among u.S. Middle and high school students from 2011-2012. 2013. Available from: http://www.cdc.gov/media/releases/2013/p0905-ecigarette-use.html.
14. Woodcock J. Letter to mr. Matt salmon, president, electronic cigarette association. 2010. Available from: http://www.fda.gov/downloads/drugs/guidancecomplianceregulatoryinformation/UCM225263.pdf.
15. World Health Organization. Questions and answers on electronic cigarettes or electronic nicotine delivery systems (ends). 2013. Available from: http://www.who.int/tobacco/communications/statements/eletronic_cigarettes/en/.
16. Dato VM. The aaphp statement on the state regulation of e-cigarettes. 2013.
17. McAuley TR, Hopke PK, Zhao J, Babaian S. Comparison of the effects of e-cigarette vapor and cigarette smoke on indoor air quality. *Inhal Toxicol* 2012;24:850-857.

18. Cahn Z, Siegel M. Electronic cigarettes as a harm reduction strategy for tobacco control: A step forward or a repeat of past mistakes? *J Public Health Policy* 2011;32:16-31.
19. Etter JF, Bullen C. Saliva cotinine levels in users of electronic cigarettes. *Eur Respir J* 2011;38:1219-1220.
20. Flouris AD, Chorti MS, Poulianiti KP, Jamurtas AZ, Kostikas K, Tzatzarakis MN, Wallace Hayes A, Tsatsakis AM, Koutedakis Y. Acute impact of active and passive electronic cigarette smoking on serum cotinine and lung function. *Inhal Toxicol* 2013;25:91-101.
21. Williams M, Villarreal A, Bozhilov K, Lin S, Talbot P. Metal and silicate particles including nanoparticles are present in electronic cigarette cartomizer fluid and aerosol. *PLoS One* 2013;8:e57987.
22. Eissenberg T. Electronic nicotine delivery devices: Ineffective nicotine delivery and craving suppression after acute administration. *Tob Control* 2010;19:87-88.
23. Aberle DR, Abtin F, Brown K. Computed tomography screening for lung cancer: Has it finally arrived? Implications of the national lung screening trial. *J Clin Oncol* 2013;31:1002-1008.
24. Hansson L, Choudry NB, Karlsson JA, Fuller RW. Inhaled nicotine in humans: Effect on the respiratory and cardiovascular systems. *J Appl Physiol (1985)* 1994;76:2420-2427.
25. Etter JF. Electronic cigarettes: A survey of users. *BMC public health* 2010;10:231.
26. Fuoco FC, Buonanno G, Stabile L, Vigo P. Influential parameters on particle concentration and size distribution in the mainstream of e-cigarettes. *Environ Pollut* 2014;184:523-529.
27. Zhang Y, Sumner W, Chen DR. In vitro particle size distributions in electronic and conventional cigarette aerosols suggest comparable deposition patterns. *Nicotine Tob Res* 2013;15:501-508.
28. Hughes JR, Goldstein MG, Hurt RD, Shiffman S. Recent advances in the pharmacotherapy of smoking. *JAMA : the journal of the American Medical Association* 1999;281:72-76.
29. Lunell E, Molander L, Ekberg K, Wahren J. Site of nicotine absorption from a vapour inhaler--comparison with cigarette smoking. *Eur J Clin Pharmacol* 2000;55:737-741.
30. McCauley L, Markin C, Hosmer D. An unexpected consequence of electronic cigarette use. *Chest* 2012;141:1110-1113.
31. Wieslander G, Norback D, Lindgren T. Experimental exposure to propylene glycol mist in aviation emergency training: Acute ocular and respiratory effects. *Occup Environ Med* 2001;58:649-655.
32. Piano MR, Benowitz NL, Fitzgerald GA, Corbridge S, Heath J, Hahn E, Pechacek TF, Howard G, American Heart Association Council on Cardiovascular N. Impact of smokeless tobacco products on cardiovascular disease: Implications for policy, prevention, and treatment: A policy statement from the american heart association. *Circulation* 2010;122:1520-1544.
33. Westenberger BJ. Letter to michael levy concerning evaluation of e-cigarettes. 2009 [cited 2013. Available from: http://www.fda.gov/downloads/drugs/scienceresearch/ucm173250.pdf.
34. Stepanov I, Jensen J, Hatsukami D, Hecht SS. Tobacco-specific nitrosamines in new tobacco products. *Nicotine Tob Res* 2006;8:309-313.
35. Burstyn I. Peering through the mist: Systematic review of what the chemistry of contaminants in electronic cigarettes tells us about health risks. *BMC public health* 2014;14:18.
36. N'Sonde V. Pas si inoffensive, la cigarette électronique ! 2013. Available from: http://www.60millions-mag.com/actualites/articles/pas_si_inoffensive_la_cigarette_electronique_nbsp.

37. Knowles MR, Paradiso AM, Boucher RC. In vivo nasal potential difference: Techniques and protocols for assessing efficacy of gene transfer in cystic fibrosis. *Human gene therapy* 1995;6:445-455.
38. Middleton PG, Caplen NJ, Gao X, Huang L, Gaya H, Geddes DM, Alton EW. Nasal application of the cationic liposome dc-chol:Dope does not alter ion transport, lung function or bacterial growth. *The European respiratory journal* 1994;7:442-445.
39. De Boeck K, Kent L, Davies J, Derichs N, Amaral M, Rowe SM, Middleton P, de Jonge H, Bronsveld I, Wilschanski M, Melotti P, Danner-Boucher I, Boerner S, Fajac I, Southern K, de Nooijer RA, Bot A, de Rijke Y, de Wachter E, Leal T, Vermeulen F, Hug MJ, Rault G, Nguyen-Khoa T, Barreto C, Proesmans M, Sermet-Gaudelus I. Cftr biomarkers: Time for promotion to surrogate end-point. *The European respiratory journal* 2013;41:203-216.
40. Wallace HL, Barker PM, Southern KW. Nasal airway ion transport and lung function in young people with cystic fibrosis. *American journal of respiratory and critical care medicine* 2003;168:594-600.
41. Dransfield MT, Wilhelm AM, Flanagan B, Courville C, Tidwell SL, Raju SV, Gaggar A, Steele C, Tang LP, Liu B, Rowe SM. Acquired cftr dysfunction in the lower airways in copd. *Chest* 2013.
42. Pruliere-Escabasse V, Fanen P, Dazy AC, Lechapt-Zalcman E, Rideau D, Edelman A, Escudier E, Coste A. Tgf-beta 1 downregulates cftr expression and function in nasal polyps of non-cf patients. *Am J Physiol Lung Cell Mol Physiol* 2005;288:L77-83.
43. Snodgrass SM, Cihil KM, Cornuet PK, Myerburg MM, Swiatecka-Urban A. Tgf-beta1 inhibits cftr biogenesis and prevents functional rescue of deltaf508-cftr in primary differentiated human bronchial epithelial cells. *PLoS One* 2013;8:e63167.
44. Rowe SM, Liu B, Hill A, Hathorne H, Cohen M, Beamer JR, Accurso FJ, Dong Q, Ordonez CL, Stone AJ, Olson ER, Clancy JP, Group VXS. Optimizing nasal potential difference analysis for cftr modulator development: Assessment of ivacaftor in cf subjects with the g551d-cftr mutation. *PLoS One* 2013;8:e66955.
45. Ramsey BW, Davies J, McElvaney NG, Tullis E, Bell SC, Drevinek P, Griese M, McKone EF, Wainwright CE, Konstan MW, Moss R, Ratjen F, Sermet-Gaudelus I, Rowe SM, Dong Q, Rodriguez S, Yen K, Ordonez C, Elborn JS, Group VXS. A cftr potentiator in patients with cystic fibrosis and the g551d mutation. *N Engl J Med* 2011;365:1663-1672.
46. Cantin AM, Hanrahan JW, Bilodeau G, Ellis L, Dupuis A, Liao J, Zielenski J, Durie P. Cystic fibrosis transmembrane conductance regulator function is suppressed in cigarette smokers. *Am J Respir Crit Care Med* 2006;173:1139-1144.
47. Solomon GM, Konstan MW, Wilschanski M, Billings J, Sermet-Gaudelus I, Accurso F, Vermeulen F, Levin E, Hathorne H, Reeves G, Sabbatini G, Hill A, Mayer-Hamblett N, Ashlock M, Clancy JP, Rowe SM. An international randomized multicenter comparison of nasal potential difference techniques. *Chest* 2010;138:919-928.
48. Bullen C, Howe C, Laugesen M, McRobbie H, Parag V, Williman J, Walker N. Electronic cigarettes for smoking cessation: A randomised controlled trial. *Lancet* 2013;382:1629-1637.
49. Etter JF, Bullen C. A longitudinal study of electronic cigarette users. *Addict Behav* 2014;39:491-494.
50. Sandberg A, Skold CM, Grunewald J, Eklund A, Wheelock AM. Assessing recent smoking status by measuring exhaled carbon monoxide levels. *PLoS One* 2011;6:e28864.
51. Jarvis MJ, Tunstall-Pedoe H, Feyerabend C, Vesey C, Saloojee Y. Comparison of tests used to distinguish smokers from nonsmokers. *Am J Public Health* 1987;77:1435-1438.
52. Standardization of spirometry, 1994 update. American thoracic society. *Am J Respir Crit Care Med* 1995;152:1107-1136.

53. Lung function testing: Selection of reference values and interpretative strategies. American thoracic society. *Am Rev Respir Dis* 1991;144:1202-1218.
54. American thoracic society. Single-breath carbon monoxide diffusing capacity (transfer factor). Recommendations for a standard technique--1995 update. *Am J Respir Crit Care Med* 1995;152:2185-2198.
55. Willemse BW, Postma DS, Timens W, ten Hacken NH. The impact of smoking cessation on respiratory symptoms, lung function, airway hyperresponsiveness and inflammation. *Eur Respir J* 2004;23:464-476.

56. Dhariwal J, Kitson J, Jones RE, Nicholson G, Tunstall T, Walton RP, Francombe G, Gilbert J, Tan AJ, Murdoch R, Kon OM, Openshaw PJ, Hansel TT. Nasal Lipopolysaccharide Challenge and Cytokine Measurement Reflects Innate Mucosal Immune Responsiveness. PLoS One 2015; 10: e0135363.
