# Supplementary material for: Differences in vaping topography in relation to adherence to exclusive electronic cigarette use in veterans
Source: PLoS One. 2018 Apr 25;13(4):e0195896. doi: 10.1371/journal.pone.0195896 (PMC5919012; doi:10.1371/journal.pone.0195896)
Supplement: S1 Table — (DOCX) [file pone.0195896.s003.docx]

**S1 Table. Demographic characteristics comparing subjects who failed and succeeded switching to EC during the Replacement and Maintenance Phases.**

|  | | **ALL** | **REPLACEMENT PHASE** | | | **MAINTENANCE PHASE** | | |
| --- | --- | --- | --- | --- | --- | --- | --- | --- |
|  |  |  | **EARLY FAILURE** | **COMPLETED** | ***p*** | **LATE FAILURE** | **COMPLETED** | ***p*** |
| **N** | | 25 | 9 | 16 |  | 5 | 8 |  |
| Age, years | | 57.8 ± 5.3 | 58 ± 4.7 | 57 ± 5.5 | 0.91 | 60.8 ± 8.1 | 56.3 ± 4.1 | 0.21 |
| Gender, %male | | 96 | 88.89 | 100 | 0.14 | 100 | 100 |  |
| Race % | African American | 80 | 55.6 | 87.5 | 0.2 | 85.7 | 87.5 | 0.24 |
|  | Hispanic | 10 | 22.2 | 6.25 |  | 14.3 | 0 |  |
|  | Caucasian | 10 | 22.2 | 6.25 |  | 0 | 12.5 |  |
| **Smoking history** | |  |  |  |  |  |  |  |
| Pack-years | | 49.8 ± 31.4 | 55.6 ± 38.4 | 46.8 ± 27.6 | 0.52 | 52 ± 35.5 | 43.2 ± 26.3 | 0.61 |
| # cigarettes per day | | 15.1 ± 8.6 | 18.7 ± 13.1 | 13.5 ± 6 | 0.23 | 15.6 ± 5.1 | 12.1 ± 6.6 | 0.38 |
| **Cotinine levels (Baseline)** | | 499.6 ± 256.8 | 542.5 ± 269.2 | 469.9 ± 254.5 | 0.5 | 517.4 ± 214.8 | 440.3 ± 286.5 | 0.61 |
| **History of Mental Illness %** | | 90 | 87.5 | 91.6 | 0.7 | 83.3 | 100 | 0.29 |
| **History of Drug Abuse %** | | 90 | 87.5 | 91.6 | 0.7 | 16.7 | 100 | 0.29 |
| **Psychological Assessments** | |  |  |  |  |  |  |  |
| CDS | | 37.4 ± 16.5 | 32.5 ± 17.7 | 39.9 ± 4.14 | 0.31 | 27 ± 17.7 | 44.2 ± 13.9 | 0.08 |
| FTND | | 5.8 ± 1.6 | 6.6 ± 1.9 | 5.5 ± 1.5 | 0.13 | 5.6 ± 1.3 | 5.5 ± 1.9 | 0.92 |
| BAI * | | 5 (0-18.7) | 3 (0-17.5) | 9 (0.5-22.5) | 0.39 | 10 (9-15) | 5 (0-30) | 0.56 |
| BDI * | | 74.5 (0-24.5) | 7 (0.5-28) | 2 (0-22.5) | 0.57 | 2 (0-12) | 0 (0-33) | 1.00 |
| HADS A * | | 5 (0.5-9) | 9 (8.2-9.7) | 3 (0-7) | 0.04 | 3 (0-8) | 2 (0-13) | 1.00 |
| HADS D * | | 3 (1-7.5) | 7.5 (3-9) | 2 (0.5-5.5) | 0.15 | 1 (1-3) | 2 (0-15) | 1.00 |
| PTSD | | 92.1 ± 31.2 | 101.2 ± 16.1 | 89.1 ± 34.2 | 0.51 | 75 ± 14.1 | 97 ± 42.3 | 0.50 |
| **Pulmonary Function** | |  |  |  |  |  |  |  |
| FEV_1_ (L) | | 2.9 ± 0.4 | 2.8 ± 0.47 | 2.9 ± 0.39 | 0.61 | 2.5 ± 0.3 | 3.18 ± 0.3 | 0.01 |
| FEV_1_ % | | 78.7 ± 10.5 | 84.8 ± 13.1 | 75.6 ± 9.1 | 0.057 | 66.2 ± 8 | 81.2 ± 6.2 | 0.00 |
| FEV_1_/FVC | | 76 ± 7.1 | 76.6 ± 9.8 | 75.7 ± 5.4 | 0.77 | 77.2 ± 4 | 73.8 ± 6.1 | 0.31 |
| FEF25-75 | | 2.45 ± 0.77 | 2.6 ± 1.09 | 2.3 ± 0.56 | 0.52 | 2 ± 0.5 | 2.5 ± 0.5 | 0.16 |
| FEF25-75% | | 69.6 ± 21.6 | 78.3 ± 31.82 | 65.31 ± 14.59 | 0.17 | 56.4 ± 13.1 | 68.3 ± 14.1 | 0.15 |

Results are expressed as Mean ± standard deviation. *Nonparametric variables: Median (IQR) (Wilcoxon/Kruskal-Wallis Test)

*CDS: Cigarette Dependence Scale, FTND: Fargestrom Test for Nicotine Dependence, BAI: Beck Anxiety Inventory, BDI: Beck Depression Inventory, HADS A: Hospital Anxiety and Depression Scale Anxiety, HADS D: Hospital Anxiety and Depression Scale Depression, PTSD: Post Traumatic Stress Disorder, FEV1: Forced Expiratory Volume in One Second, FEV1/FVC: Forced Expiratory Volume in One Second/Forced Vital Capacity ratio, FEF: Forced Expiratory Flow*
