# Supplementary material for: Differences in vaping topography in relation to adherence to exclusive electronic cigarette use in veterans
Source: PLoS One. 2018 Apr 25;13(4):e0195896. doi: 10.1371/journal.pone.0195896 (PMC5919012; doi:10.1371/journal.pone.0195896)
Supplement: S2 Table — (DOCX) [file pone.0195896.s004.docx]

**S2 Table. Vaping patterns of Replacement Phase and Maintenance Phase by Groups**

|  | | **Replacement Phase** | **Maintenance Phase** | ***p*** |
| --- | --- | --- | --- | --- |
| **Vapes per Day** | Success Group | 139.4 ± 138.0 | 218.0 ± 173.3 | 0.02 |
|  | Failure Group | 114.6 ± 94.0 | 159.9 ± 76.7 | 0.03 |
| **Voltage per Vape** | Success Group | 3.8 ± 0.76 | 4.3 ± 0.75 | <0.01 |
|  | Failure Group | 3.8 ± 0.74 | 3.6 ± 0.67 | 0.43 |
| **Wattage per Vape** | Success Group | 10.0 ± 4.14 | 12.1 ± 4.2 | 0.02 |
|  | Failure Group | 9.9 ± 3.9 | 9.4 ± 2.0 | 0.53 |
| **Seconds per Vape** | Success Group | 5.7 ± 1.4 | 6.1 ± 1.3 | 0.10 |
|  | Failure Group | 3.7 ± 1.5 | 4.4 ± 1.9 | 0.06 |
| **Vape-Seconds** | Success Group | 698.7 ± 651.9 | 1259.7 ± 1037.8 | <0.01 |
|  | Failure Group | 367.5 ± 284.0 | 622.3 ± 295.8 | <0.01 |
