# Supplementary figures and images for: Differences in vaping topography in relation to adherence to exclusive electronic cigarette use in veterans
Source: PLoS One. 2018 Apr 25;13(4):e0195896. doi: 10.1371/journal.pone.0195896 (PMC5919012; doi:10.1371/journal.pone.0195896)

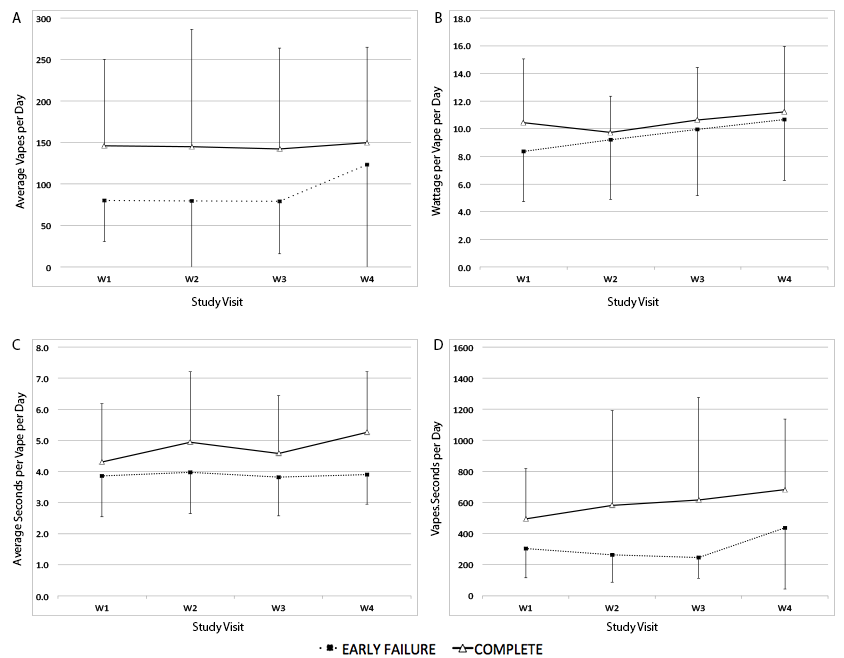

Supplement: S1 Fig — This graph includes all subjects that entered the replacement phase. Complete = subjects who successfully replaced tobacco smoking with electronic cigarette in a 4-week period. Early failure = subjects who could not replace tobacco smoking with electronic cigarette. Comparison between groups was statistically significant for number of vapes per day (p = 0.01), voltage (p = 0.01) and vapes-seconds (p<0.001). (TIFF) [file pone.0195896.s005.tiff]

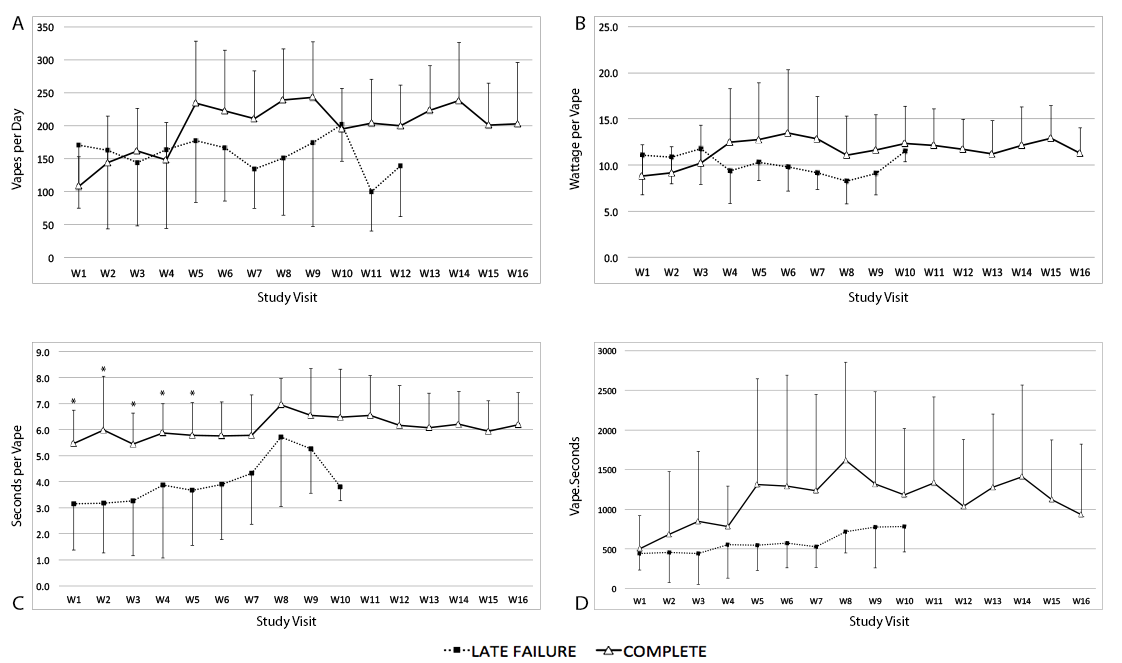

Supplement: S2 Fig — This graph includes all subjects who entered the maintenance phase (successfully replaced TC smoking to EC vaping, N = 16). Complete = subjects who successfully continued on exclusive EC use in a 12-week period. Late failure = subjects who relapsed to TC smoking. (TIF) [file pone.0195896.s006.tif]
